# The chromosome-level genome of Chinese praying mantis *Tenodera sinensis* (Mantodea: Mantidae) reveals its biology as a predator

**DOI:** 10.1093/gigascience/giad090

**Published:** 2023-10-26

**Authors:** Ruizhong Yuan, Boying Zheng, Zekai Li, Xingzhou Ma, Xiaohan Shu, Qiuyu Qu, Xiqian Ye, Sheng Li, Pu Tang, Xuexin Chen

**Affiliations:** Institute of Insect Sciences, College of Agriculture and Biotechnology, Zhejiang University, Hangzhou 310058, China; State Key Lab of Rice Biology, Ministry of Agriculture Key Lab of Molecular Biology of Crop Pathogens and Insects, and Zhejiang Provincial Key Laboratory of Biology of Crop Pathogens and Insects, Zhejiang University, Hangzhou 310058, China; Institute of Insect Sciences, College of Agriculture and Biotechnology, Zhejiang University, Hangzhou 310058, China; State Key Lab of Rice Biology, Ministry of Agriculture Key Lab of Molecular Biology of Crop Pathogens and Insects, and Zhejiang Provincial Key Laboratory of Biology of Crop Pathogens and Insects, Zhejiang University, Hangzhou 310058, China; Institute of Insect Sciences, College of Agriculture and Biotechnology, Zhejiang University, Hangzhou 310058, China; State Key Lab of Rice Biology, Ministry of Agriculture Key Lab of Molecular Biology of Crop Pathogens and Insects, and Zhejiang Provincial Key Laboratory of Biology of Crop Pathogens and Insects, Zhejiang University, Hangzhou 310058, China; Institute of Insect Sciences, College of Agriculture and Biotechnology, Zhejiang University, Hangzhou 310058, China; State Key Lab of Rice Biology, Ministry of Agriculture Key Lab of Molecular Biology of Crop Pathogens and Insects, and Zhejiang Provincial Key Laboratory of Biology of Crop Pathogens and Insects, Zhejiang University, Hangzhou 310058, China; Institute of Insect Sciences, College of Agriculture and Biotechnology, Zhejiang University, Hangzhou 310058, China; State Key Lab of Rice Biology, Ministry of Agriculture Key Lab of Molecular Biology of Crop Pathogens and Insects, and Zhejiang Provincial Key Laboratory of Biology of Crop Pathogens and Insects, Zhejiang University, Hangzhou 310058, China; Hainan Institute, Zhejiang University, Sanya 572025, China; Institute of Insect Sciences, College of Agriculture and Biotechnology, Zhejiang University, Hangzhou 310058, China; State Key Lab of Rice Biology, Ministry of Agriculture Key Lab of Molecular Biology of Crop Pathogens and Insects, and Zhejiang Provincial Key Laboratory of Biology of Crop Pathogens and Insects, Zhejiang University, Hangzhou 310058, China; Hainan Institute, Zhejiang University, Sanya 572025, China; Institute of Insect Sciences, College of Agriculture and Biotechnology, Zhejiang University, Hangzhou 310058, China; State Key Lab of Rice Biology, Ministry of Agriculture Key Lab of Molecular Biology of Crop Pathogens and Insects, and Zhejiang Provincial Key Laboratory of Biology of Crop Pathogens and Insects, Zhejiang University, Hangzhou 310058, China; Guangdong Provincial Key Laboratory of Insect Developmental Biology and Applied Technology, Institute of Insect Science and Technology, School of Life Sciences, South China Normal University, Guangzhou 510631, China; Guangmeiyuan R&D Center, Guangdong Provincial Key Laboratory of Insect Developmental Biology and Applied Technology, South China Normal University, Meizhou 514779, China; Institute of Insect Sciences, College of Agriculture and Biotechnology, Zhejiang University, Hangzhou 310058, China; State Key Lab of Rice Biology, Ministry of Agriculture Key Lab of Molecular Biology of Crop Pathogens and Insects, and Zhejiang Provincial Key Laboratory of Biology of Crop Pathogens and Insects, Zhejiang University, Hangzhou 310058, China; Institute of Insect Sciences, College of Agriculture and Biotechnology, Zhejiang University, Hangzhou 310058, China; State Key Lab of Rice Biology, Ministry of Agriculture Key Lab of Molecular Biology of Crop Pathogens and Insects, and Zhejiang Provincial Key Laboratory of Biology of Crop Pathogens and Insects, Zhejiang University, Hangzhou 310058, China; Hainan Institute, Zhejiang University, Sanya 572025, China

**Keywords:** Mantodea, *Tenodera sinensis*, chromosome, insect genomics, mantis, digestive demand, predation behavior

## Abstract

**Background:**

The Chinese praying mantis, *Tenodera sinensis* (Saussure), is a carnivorous insect that preys on a variety of arthropods and small vertebrates, including pest species. Several studies have been conducted to understand its behavior and physiology. However, there is limited knowledge about the genetic information underlying its genome evolution, digestive demands, and predatory behaviors.

**Findings:**

Here we have assembled the chromosome-level genome of *T. sinensis*, representing the first sequenced genome of the family Mantidae, with a genome size of 2.54 Gb and scaffold N50 of 174.78 Mb. Our analyses revealed that 98.6% of BUSCO genes are present, resulting in a well-annotated assembly compared to other insect genomes, containing 25,022 genes. The reconstructed phylogenetic analysis showed the expected topology placing the praying mantis in an appropriate position. Analysis of transposon elements suggested the Gypsy/Dirs family, which belongs to long terminal repeat (LTR) transposons, may be a key factor resulting in the larger genome size. The genome shows expansions in several digestion and detoxification associated gene families, including trypsin and glycosyl hydrolase (GH) genes, ATP-binding cassette (ABC) transporter, and carboxylesterase (CarE), reflecting the possible genomic basis of digestive demands. Furthermore, we have found 1 ultraviolet-sensitive opsin and 2 long-wavelength-sensitive (LWS) opsins, emphasizing the core role of LWS opsins in regulating predatory behaviors.

**Conclusions:**

The high-quality genome assembly of the praying mantis provides a valuable repository for studying the evolutionary patterns of the mantis genomes and the gene expression profiles of insect predators.

## Introduction

Mantodea, an order of insects, belongs to the larger Polyneoptera group. This diverse group encompasses a wide array of species, including grasshoppers, crickets, and even cockroaches, each boasting their own adaptations and behaviors [1–3]. Mantodea currently comprises 29 extant families and 3 fossil families, with almost 3,000 known extant species [4]. All mantis species are predators and feed on other organisms from the moment they hatch and in populations where space is limited, and cannibalism is common, with females even consuming males on occasion [5, 6]. Species in the order Mantodea, particularly those within the family Mantidae, are ambush predators [7].

**Figure 1:**
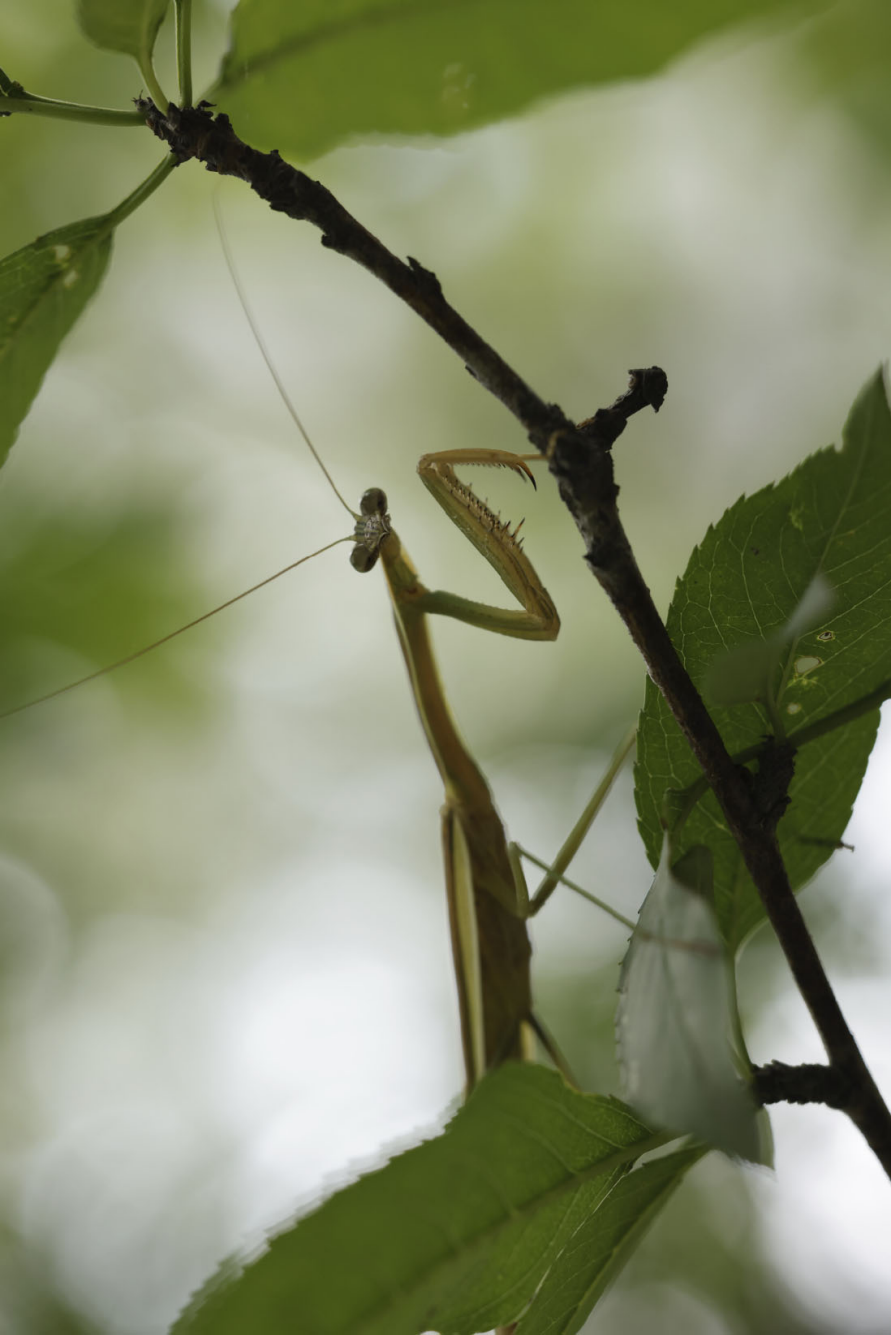
The Chinese praying mantis (*Tenodera sinensis*) whose genome was sequenced, at the Institute of Insect Sciences, Zhejiang University (Hangzhou, China). Photo by Xiqian Ye.

*Tenodera sinensis* Saussure (Mantodea: Mantidae; NCBI:txid406589) (Fig. 1), commonly known as the Chinese praying mantis, is widely distributed in China [8]. Due to its predatory nature, it has great biological control potential, which makes it an important species for pest management. Unlike other feeding insects, the praying mantis requires the digestion of higher amounts of protein and the metabolism of various toxins from venomous prey [9, 10]. Moreover, the praying mantis has a highly specialized visual system that allows it to detect and track prey with incredible accuracy, and its compound eyes have a wide field of view and can detect both color and motion [11–13]. Additionally, the praying mantis has an ability to perceive depth, which is essential for accurately striking and capturing prey [14]. However, despite the importance of the praying mantis in pest management, there is limited information on relevant gene families because there have been only a few studies on its digestive demand and vision ability. Genetic deciphering of the praying mantis provides valuable data and clues for understanding the gene expression profiles of predators.

High-quality chromosome-level genomes of mantis are essential for understanding the biological information of insect predators. However, due to issues such as large genome size, and genetic diversity caused by different geographical populations, high-quality genomic data for Mantodea are currently unavailable. In this study, we assembled the genome of *T. sinensis* at the chromosomal level by combining Illumina, PacBio (single-molecule real-time sequencing) and Hi-C (high-throughput chromosome conformation capture) sequencing. The assembly of the genome provides valuable genomic resources for researchers studying insect predators, aiding in the development of biological control strategies, population genetics, and evolutionary and phylogeny studies of insect genomes. Overall, the high-quality mantis genome assembly will undoubtedly have a significant impact on the field of entomology and related research areas.

## Results

### Genome assembly

The genome survey of *T. sinensis* was initially found to have a low level of heterozygosity (0.47%) in a large 2.42-Gb genome (Supplementary Fig. S1, Supplementary Table S1). Given the large size of the genome, assembling a chromosome-level genome may prove to be challenging. To assemble a high-quality genome, a combination of PacBio long-read and Illumina short-read sequencing was used. The primary genome assembly was based on the data after quality control (467.29 Gb) generated by the PacBio Sequel I platform. Subsequently, the *de novo* assembly of the long-read data obtained from PacBio sequencing was polished and improved using the next-generation sequencing (NGS) data (23.16 Gb). The resulting reference genome assembly for *T. sinensis* has a total length of 2.45 Gb, comprising 1,763 scaffolds and 1,820 contigs with N50 lengths of 3.07 Mb and 3.05 Mb, respectively.

**Figure 2:**
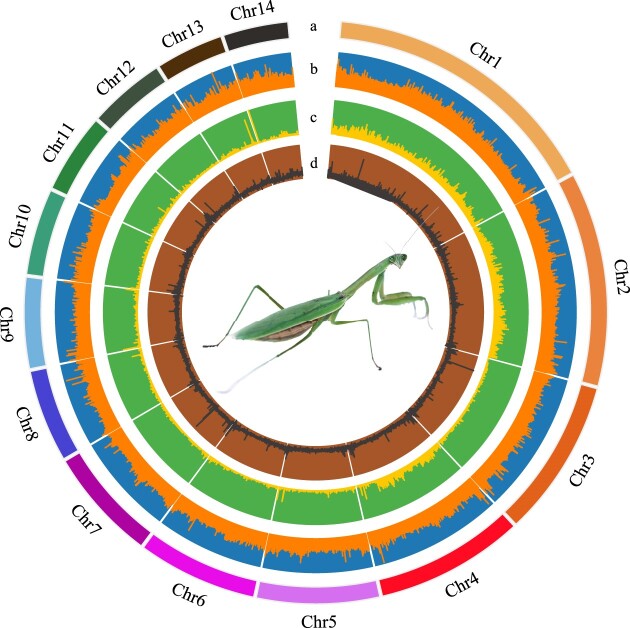
Genome assemble of *Tenodera sinensis*. “a” means chromosome ID, “b” means GC content, “c” means repetitive sequence content, and “d” means gene content.

The reference assembly of *T. sinensis* was further improved using Hi-C analysis with 315.96 Gb Hi-C data. Using the contig interaction frequency calculated from the alignment of pairs with contigs, 96.45% of reference genome sequences were found to be successfully anchored in 14 pseudochromosome groups (Supplementary Fig. S2). This completion marked the first ever chromosome-level genome of Mantidae, with a genome size of 2.54 Gb and a scaffold N50 length of 174.78 Mb (Table 1). The quality of the final genome was assessed using BUSCO v5.2.2 with the insect_obd10 database (Table 1, Supplementary Table S3) and arthropoda_obd10 database (Supplementary Table S4). The analysis identified 98.6% of highly conserved insect genes, indicating that the assembled *T. sinensis* genome is of high quality and can be used for further functional and comparative genomics studies (Fig. 2). In order to interrogate the *T. sinensis* genome assembly, we aligned each read of the genome to the NT library using BLAST. Out of the 2,189 reads from the final assembled genome, 1,606 very short debris reads (73.77% of the reads) were not compared, but among the reads that were compared to the database, 90.90% were related to arthropods, and 92.45% were related to insects (Supplementary Fig. S3). While the debris reads were numerous, they did not contribute significantly to the overall genome size. For the 14 chromosomes that accounted for 96.45% of the size, we aligned 14 chromosomes to the NT library. Among them, 64.29% were related to *T. sinensis*, 21.43% to *Timema bartmani*, 7.14% to *Onthophagus taurus*, and 7.14% to *Timema tahoe*, suggesting that the final assembly does not contain any sequences of nontarget organisms from contaminants/symbionts in the DNA library (Supplementary Fig. S4).

**Table 1:** Genome assembly of *Tenodera sinensis*. "C" means complete BUSCOs, "S" means complete and single-copy BUSCOs, "D" means complete and duplicated BUSCOs, "F" means fragmented BUSCOs, "M" means missing BUSCOs and "n" means the number of gene in BUSCO gene set. .

Species	*Tenodera sinensis*
Lineage	Mantodea: Mantidae
Genome level	Chromosome
Genome size (Mb)	2,537.11
GC content (%)	37.62
Sequence number	2189
Contig N50 (Mb)	2.36
Scaffold N50 (Mb)	174.78
Maximum scaffold length (bp)	422,258,719
Minimum scaffold length (bp)	1,000
Complete BUSCO score—Insecta	C :98.6% [S: 95.2%, D: 3.4%], F: 0.5%, M: 0.9%, n: 1,367
Complete BUSCO score—Arthropoda	C: 99.0%[S: 95.8%, D: 3.2%], F: 0.4%, M: 0.6%, n: 1,013

### Genome annotation

A combination of *de novo*, transcriptome data and homology-based methods was used for predicting gene models. The genome was found to have a total of 1.78 Gb repetitive sequences, which accounted for 72.85% of the genome. The GC content of the genomic contigs was 37.62% (Supplementary Table S5). Among the repetitive sequences, DNA transposons and long interspersed nuclear elements (LINEs) were found to be the most predominant, accounting for 36.63% and 10.77%, respectively (Supplementary Table S2).

Gene functional annotation is helpful for understanding the complex relationship between internal genes and external traits. The models of protein-coding genes were identified using *de novo* and homology-based prediction methods based on the transcriptome data, resulting in the identification of 25,022 protein-coding genes. Furthermore, functional annotation was performed to identify 19,521 GO terms, 5,363 KEGG ko terms, 2,104 enzyme codes, 758 KEGG pathways, and 3,478 clusters of orthologous groups (COGs) categories (Table 2, Supplementary Tables S6–S10). Besides protein-coding genes, noncoding RNAs (ncRNAs) were also identified as important regulatory components in gene expression and epigenetics. Six types of ncRNAs were identified, including 482 ribosomal RNAs (rRNAs), 139 microRNAs (miRNAs), 336 small nuclear RNAs (snRNAs), 80,972 transfer RNAs (tRNAs), 2 small RNAs (sRNAs), and 2 long noncoding RNAs (lncRNAs) (Table 2, Supplementary Table S11).

**Table 2:** Genome annotation of *Tenodera sinensis*. The numbers in the “Functional annotation” column represent the count of genes that have been matched or have hits in different databases for functional annotation.

Structural annotation	
Genes	25,022
Mean gene length (bp)	34,430
Repeat (%)	72.85
Noncoding RNAs	
rRNA	482
miRNA	139
snRNA	336
tRNA	80,972
sRNA	2
lncRNA	2
Functional annotation	
Nr	10,788
Swiss-prot	7,851
TrEMBL	10,715
Interproscan	15,560
EggNOG	10,523
Unannotated	34.69%
Total annotated	65.31%

### Phylogenetic analysis

Identifying homologous relationships among sequences from different species is crucial for improving our understanding of evolution and biodiversity. In this regard, we compared the protein-coding genes of *T. sinensis* with those of 12 representatives, including 6 polyneopteran species (*Gryllus bimaculatus* [Orthoptera] [15], *Losta migratoria* [Orthoptera] [16, 17], *Clitarchus hookeri* [Phasmatodea] [18], *Blattella germanica* [Blattodea] [19], *Cryptotermes secundus* [Blattodea] [19], and *Zootermopsis nevadensis* [Blattodea] [20]) and 6 other insect species (*Ephemera danica* [Ephemeroptera] [21], *Rhodnius prolixus* [Hemiptera] [22], *Apis mellifera* [Hymenoptera] [23], *Drosophila melanogaster* [Diptera] [24], *Tribolium castaneum* [Coleoptera] [25], and *Bombyx mori* [Lepidoptera] [26]), with *Catajapyx aquilonaris* (Diplura: Japygidae) [27] being an outgroup. Using OrthoFinder, a total of 216,767 genes were analyzed among the 14 species, of which 177,216 were clustered into 16,153 orthogroups. We also analyzed the genes of single-copy and multicopy orthologs, as well as unique genes and unassigned orthologous genes for each species. In the *T. sinensis* genome, 2,436 genes with no orthology relationship are clustered in 574 unique orthologous groups when compared to the other 13 species.

**Figure 3:**
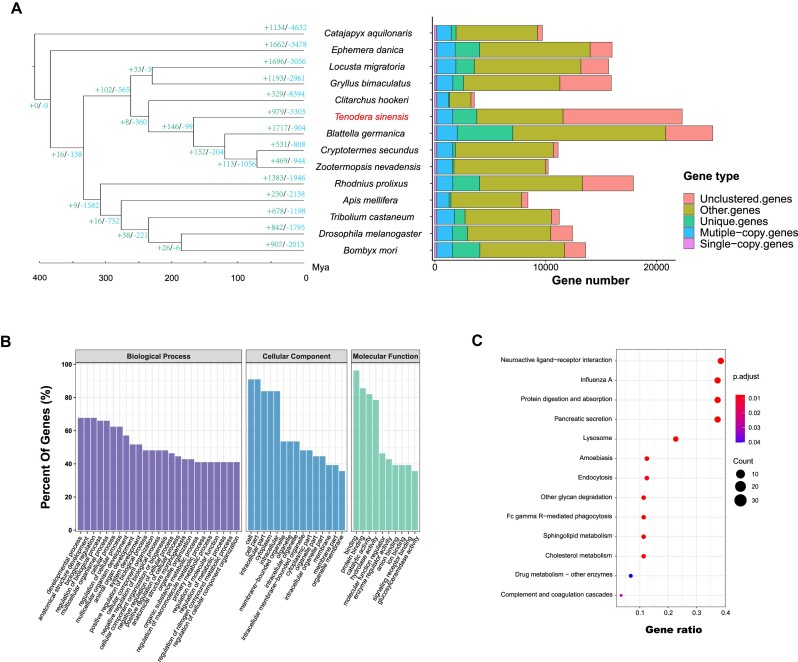
Phylogenetic analyses of *Tenodera sinensis* and GO, KEGG of rapid evolved genes in expansion. (A) Phylogenetic tree of *T. sinensis* and other 13 species. The estimated species divergence times (million years ago, Mya) are indicated at each branch point. Node values indicate gene families showing expansion (green) and contraction (blue). The bar chart indicates the number of genes classified into 5 groups (single-copy genes, multiple-copy genes, unique genes, other genes, and unclustered genes). (B) The GO enrichment of rapidly evolved genes in expansion. (C) The KEGG pathway analyses of rapidly evolved genes in expansion.

To gain an understanding of Mantodea genomic evolution, we reconstructed a phylogenomic tree of the 14 species based on 221 single-copy orthologs (1,548,781 amino acids) (Fig. 3A). The phylogenetic relationships of 14 insect species were well recovered, with all the nodes being strongly supported. Our results indicated that the ancestors of *T. sinensis* originated in the Jurassic period, around 167.19 million years ago.

To investigate the rapidly evolving orthologous groups in *T. sinensis*, we used orthologous group evolution analysis to uncover the changes that have occurred in certain orthologous groups over time. We found 979 orthologous groups had undergone expansions, while 1,723 orthologous groups had experienced contractions. Out of these, 31 orthologous groups (25 expansions and 6 contractions) were recognized as rapidly evolving orthogroups (Table 3). The significantly expanded orthologous groups were primarily associated with digestion (trypsin), detoxification (carboxylesterase, ABC transporter), glycometabolism (glycosyl hydrolase), and DNA transposition (DDE superfamily endonuclease, PiggyBac transposable element-derived protein). The significantly contracted orthologous groups mainly focus on chemoreception (odorant receptor, ionotropic glutamate receptor), and we unexpectedly found other digestive-related orthologous groups (glutathione S-transferase, fatty acyl-coenzyme A reductase) were significantly contracted in the *T. sinensis* genome.

**Table 3:** Rapidly evolving gene families during the evolution of *Tenodera sinensis*

OG number	Evolving type	Annotation
OG0000003	Expansion	Trypsin
OG0000038	Expansion	Zinc finger C2H2-type protein
OG0000040	Expansion	DDE superfamily endonuclease
OG0000099	Expansion	PIF1-like helicase
OG0000126	Expansion	Hypothetical protein
OG0000138	Expansion	Zinc finger BED-type protein
OG0000212	Expansion	DDE superfamily endonuclease
OG0000244	Expansion	Glycosyl hydrolase
OG0000275	Expansion	PiggyBac transposable element-derived protein
OG0000296	Expansion	Endonuclease-reverse transcriptase
OG0000337	Expansion	Serpin
OG0000338	Expansion	PiggyBac transposable element-derived protein
OG0000364	Expansion	Carboxylesterase type B
OG0000369	Expansion	Serpin Kazal-type
OG0000505	Expansion	Reverse transcriptase
OG0000529	Expansion	Hypothetical protein
OG0000559	Expansion	CRAL-TRIO lipid binding domain
OG0000633	Expansion	Ankyrin repeats
OG0000709	Expansion	ABC transporter
OG0001080	Expansion	Ankyrin repeats
OG0001361	Expansion	Hypothetical protein
OG0001367	Expansion	Hypothetical protein
OG0003219	Expansion	Testicular haploid expressed repeat
OG0004380	Expansion	Hypothetical protein
OG0004408	Expansion	Hypothetical protein
OG0000022	Contraction	Odorant receptor
OG0000041	Contraction	Short-chain dehydrogenase
OG0000044	Contraction	Glutathione S-transferase
OG0000047	Contraction	Ionotropic glutamate receptor
OG0000076	Contraction	Odorant receptor
OG0000095	Contraction	Fatty acyl-coenzyme A reductase

The rapidly expanded orthologous groups were further confirmed to be involved in metabolic detoxification, digestion, and secondary metabolite synthesis, as shown in the GO and KEGG enrichments (Fig. 3B, C). These results indicated that *T. sinensis* processes strong digestion and detoxification ability, which may enable it to effectively respond to toxic compounds present in its prey.

### Evolution of genome size

**Figure 4:**
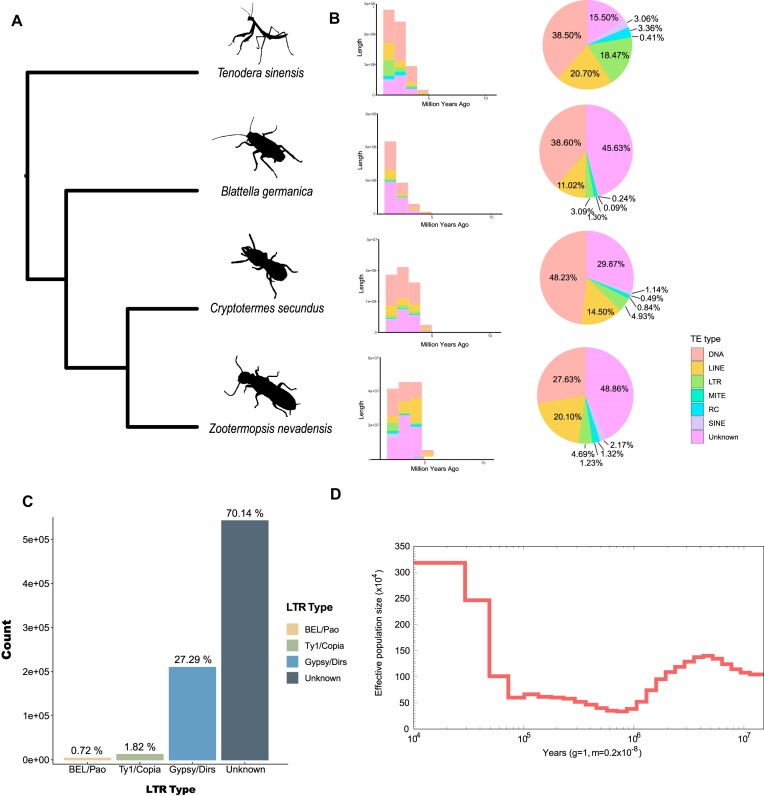
Genome size evolution. (A) The phylogenetic relationship between 4 species from Dictyoptera. (B) TE insertion time and TE content. The bar chart shows the TE insertion time and its length (bp), and the pie chart shows the percentage of different TE types. (C) The distribution of LTR type in *T. sinensis*. (D) The demographic history of *T. sinensis*. The red line represents the pairwise sequentially Markovian coalescent (PSMC) estimate. The plot was constructed assuming a generation time of 1.00 years and mutation rate of 0.2 × 10^−9^ per generation.

The expansion of DDE superfamily endonuclease and PiggyBac transposable element-derived protein indicates the high activity of transposons in the *T. sinensis* genome, which may lead to large-scale genome duplication in the ancestry of Mantodea (Table 3). Analysis of transposon element (TE) types and the TE insertion time in 4 species in Dictyoptera showed significant differences in TE content and concentration of TE insertion times in the past 5 million years in Dictyoptera (Fig. 4A, Supplementary Tables S12–S15). However, the total length of TEs in the *T. sinensis* genome is about 1.8 to 3.3 times more than others, and the proportion of long terminal repeat (LTR) content (∼18.47%) is much higher, suggesting that the recent outbreak of LTRs may have caused a large-scale enlargement in the evolutionary process of *T. sinensis* and driven the enlargement of its genome size (Fig. 4B). It is noteworthy that the proportion of LTR retrotransposons in the *T. sinensis* genome is higher than in the other 3 insects in Dictyoptera (Fig. 4B). We observed that the Gypsy/Dirs family is the predominant LTR type in the *T. sinensis* genome (Fig. 4C), and most LTR retrotransposons are short length (<2,000 bp) (Supplementary Fig. S5). The Gypsy/Dirs family can duplicate themselves within the genome and insert into new locations, resulting in changes in genome structure and function [28–30], which may be a key factor contributing to the large genome size of *T. sinensis* and its evolution. Furthermore, demographic analysis shows that the effective population size of *T. sinensis* tends to have a large fluctuation (Fig. 4D), with a growth of population size about 100,000 years ago.

### Characteristic digestive demand in insect predators

Mantodea, a member of Dictyoptera, is different from other feeding insects in its predatory characteristics, including high digestive demand and detoxification capabilities. It was observed in the*T. sinensis* genome that trypsin and glycosyl hydrolase (GH) showed expansion, while fatty acyl-coenzyme A reductase (FacR) showed contraction, indicating that *T. sinensis* has a strong ability to digest and metabolize proteins, sugars, and lipids (Supplementary Tables S16–S18). Furthermore, expansion was observed in the ATP-binding cassette (ABC) transporter and carboxylesterase (CarE), while the glutathione S-transferase (GST) showed contraction, which may indicate predatory insects rely heavily on the detoxification gene family (Supplementary Tables S19–S21). To gain further insight into the digestive demand proteins and detoxification characteristics of predatory insects, a comparison was made between the protein families of *T. sinensis* and those of omnivorous cockroaches and scavenging termites in Dictyoptera.

The praying mantis primarily feeds on insects that are rich in protein, which explains the number of trypsin and GHs found in the mantis genome compared to other insects. We identified 107 trypsin coding genes and 11 GHs in the *T. sinensis* genome, the highest numbers among Dictyopteran species. However, while the amount of trypsin in mantis is only slightly more abundant than in cockroaches and termites, it suggests that different pancreatic proteins may perform the function of protein digestion in different feeding insects. Interestingly, even though the number of FacRs has expanded in the *T. sinensis* genome, it is still lower compared to the cockroach and termites, suggesting that during the predatory evolution of *T. sinensis*, multiple copies of trypsin genes and GHs are present in the genome due to the great demand for protein digestion. Nonetheless, it emphasizes that even though the demand for lipid digestion may be less noticeable, FacRs still performs an indispensable function of protein digestion in insect predators.

**Figure 5:**
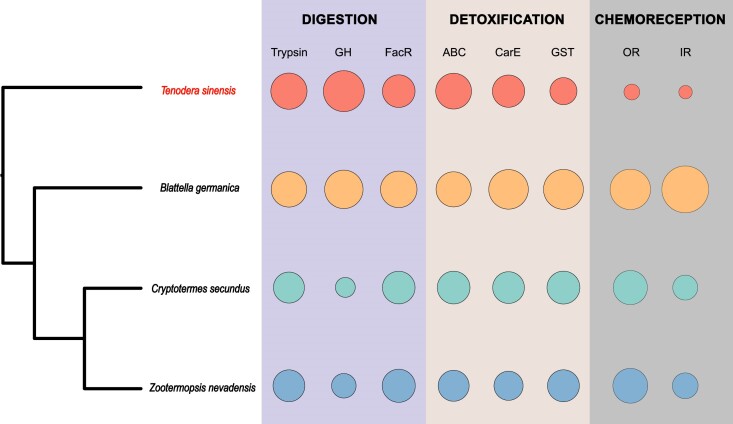
Expansion and contraction of digestion, detoxification, and chemoreception gene families in Dictyoptera. The size of the circle represents the gene count of each gene family, and 4 species in Dictyoptera are counted, *T. sinensis* (red), *B. germanica* (orange), *C. secundus* (slight blue), and *Z. nevadensis* (blue).

The detoxification gene family, such as P450, ABC transporter gene family, and carboxylesterase, plays a crucial role in insect feeding, digestion, and metabolism. The ABC transporter gene family, one of the largest protein families that exists at all stages of life, acts as major active transporters, hydrolyzing ATP to transport toxic metabolites across membranes [31]. Similarly, CarE, an essential metabolic detoxification enzyme, is mainly involved in the hydrolysis of compounds containing ester bonds inside an organism's body [32]. It can metabolize and degrade harmful substances, preventing them from reaching target sites [33, 34]. In the *T. sinensis* genome, we observed an expansion of ABC transporter and carboxylesterase genes. We annotated 77 ABC transporter genes and 45 carboxylesterase genes (Fig. 5), indicating that the praying mantis has a strong detoxification ability, while the toxins in the praying mantis mostly come from the external environmental toxins and the internal toxins present in the prey organisms.

**Figure 6:**
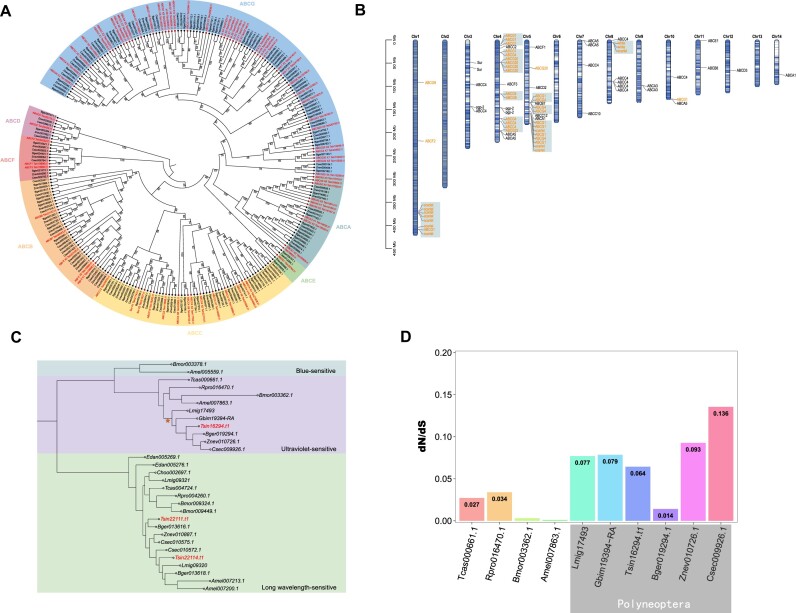
Analysis of ABC transport genes and visual genes. (A) Expansion of the ABC transport gene family in *Tenodera sinensis*. The phylogenetic tree shows the orthologous and paralogous relationship of all 259 ABC transport genes from *T. sinensis* and the other 3 species in Dictyoptera. Seven clades are marked as subfamilies, and gene labels of *T. sinensis* are marked in red. Bootstrap values are indicated on the node. (B) The distribution of the ABC transport genes in *T. sinensis*. The ABCG subfamily genes are marked in orange. (C) The phylogenetic tree of visual genes from *T. sinensis* and the other 13 species. Three clades are marked as long wavelength sensitive (green), ultraviolet sensitive (purple), and blue sensitive (blue). The dN/dS (ω) values are indicated in the visual genes in *T. sinensis*. (D) The dN/dS (ω) values of 6 species from Polyneoptera and the other 4 species.

Our phylogenetic analysis revealed a distinct expansion in the ABCG gene subfamily within the ABC transporter gene family (Fig. 6A), which is consistent with previous studies revealing that ABCG genes in arthropods play a crucial role in eliminating cholesterol from the body [35, 36]. It is possible that ABCG genes in the praying mantis also perform a similar function. To investigate this further, we plotted the locations of the ABC gene family on chromosomes and identified that the ABCG gene subfamily was mainly distributed across chromosome 1 (Chr1), chromosome 4 (Chr4), chromosome 5 (Chr5), and chromosome 8 (Chr8) (Fig. 6B). We also observed that the ABCG gene subfamily genes occurred in clusters on Chr1, Chr4, Chr5, and Chr8. Furthermore, both the *white* gene and *scarlet* gene, as members of the ABCG gene subfamily, were found in clusters on Chr8, and the *scarlet* gene was found in clusters on Chr1, suggesting that the ABCG gene subfamily may have produced multiple copies through gene replication events during evolution. The presence of multiple copies of the ABCG gene subfamily indicates that it performs an important function in the praying mantis, which is possibly related to the complex dietary mechanisms of insect predators, while *white* genes and *scarlet* genes could play a vital role in detoxification alongside the ABCG gene subfamily.

### Chemoreception and vision genes involved in insect predatory behaviors

Chemoreception performs crucial functions in predatory behaviors. Insects rely on 2 major gene families, odorant receptors (ORs) and ionotropic receptors (IRs), to mediate their sense of smell and taste. Our research has revealed a significant reduction in the number of ORs and IRs in the *T. sinensis* genome (Fig. 5, Table 3, Supplementary Tables S22–S23). This suggests that while olfaction plays an important role in locating prey habit [37], the predation behavior of the mantis may not primarily rely on the regulation of the olfactory system. Instead, it may be more influenced by vision or other sensation mechanisms.

Throughout their evolution, most mantises have transitioned from active hunters to predators that ambush their prey. *T. sinensis*, for example, primarily waits for prey by hiding in the stems and leaves of low plants at dusk, relying on their visual ability to locate prey. Electroretinography studies have suggested that the praying mantis possesses a vision with major peak sensitivity to the “green” region of the spectrum [38]. We identified 3 opsins in the *T. sinensis* genome, including 1 ultraviolet-sensitive (UV-sensitive) opsin and 2 long-wavelength-sensitive (LWS) opsins (Supplementary Table S24). The more LWS opsins that were identified may explain why the praying mantis has major peak sensitivity in the “green” region in the spectrum, while also emphasizing the core role of LWS opsins in regulating the prey behavior of *T. sinensis*. Overall, these results suggest that the praying mantis possesses at least 2 kinds of opsins at least, enabling them to distinguish colors in nature. This ability may cause *T. sinensis* to prey on insects that have more varied appearances in terms of color [39], while also making it easier for their hunting behaviors to be affected by disruptive or warning coloration in longer wavelength light.

We combined the opsins of Polyneoptera with those of representative species in Holometabola to test the phylogenetic relationship (Fig. 6C). Our analysis revealed that the UV-sensitive opsin of Polyneoptera formed a distinct clade, indicating the independent evolution of UV-sensitive opsin in this group. Subsequently, we analyzed the rate ratio (ω) of nonsynonymous to synonymous nucleotide substitution rate (Ka/Ks ratio) of each UV-sensitive opsin and found that the UV-sensitive opsin of *T. sinensis* generally had lower Ka/Ks ratios compared to those of species in Polyneoptera (Fig. 6D). It suggests that the UV-sensitive opsin in *T. sinensis* evolved at a slower rate compared to other polyneopteran species.

## Materials and Methods

### Sampling

The line of *Tenodera sinensis* was supported by the Lisheng Zhang team of the Chinese Academy of Agricultural Science. The rearing temperature was set at 23.5°C, the humidity was set at 80%, and fruit flies and mealworms were used to feed the cultivation for multiple generations. Morphological identification and mitochondrial genome information both showed that the species was *T. sinensis*. An adult female *T. sinensis* was used for the genome sequencing.

### Genome sequencing and survey

The genomic DNA was extracted using the FastPure Cell/Tissue DNA Isolation Mini Kit (Vazyme Biotech Co., Ltd.) according to the manufacturer's instructions. Whole-genome shotgun sequencing was performed using the PacBio and Illumina NovaSeq 6000 (RRID:SCR_016387) sequencing platforms. A single-molecule real-time (SMRT) bell library was constructed and run on a SMRT cell in the PacBio Sequel I system (Pacific Biosciences), generating a total of 467.29 Gb raw data. The sequencing depth from the PacBio sequencing platform was about 94.49×. The paired-end sequencing raw data generated by the Illumina sequencing platform were 23.16 Gb, and its sequencing depth was about 9.34×.

Illumina paired-end sequenced raw reads for the genomic survey were filtered using the Fastp v0.20.1 (RRID:SCR_016962) [40] preprocessor (set to default parameter) to remove low-quality reads, adapters, and reads containing poly-N. The size of the *T. sinensis* genome was estimated by a *k*-mer analysis of the Illumina clean reads. The clean reads in the NGS paired-end libraries were subjected to 21-mer frequency distribution analysis as follows: Genome size = (K-mernum/main peak depth) × (1 − Error rate). The *k*-mer profile was thus generated using Jellyfish v2.2.10 (RRID:SCR_005491) [41] to calculate the *k*-mer number and distribution, and the content of repeated sequences and the heterozygosity were calculated by GenomeScope v1.00 (RRID:SCR_017014) [42].

### Genome assembly

The primary assembly of the clean subreads from the PacBio platform was carried out using nextDenovo v2.5.0 [43] and corrected using Canu v2.1.1 (RRID:SCR_015880) [44]. The Illumina data were further used to polish and improve the genome assembly using nextPolish v1.4.0 [45]. The haplotigs and contig overlaps in a *de novo* assembly were removed using purge dups v1.2.5 (RRID:SCR_021173) [46] based on read depth. The quality of the reference genome sequence was evaluated using BUSCO v5.2.2 (RRID:SCR_015008) [47]. Finally, we aligned the final assembled genome sequence to the NT library to interrogate the genome assembly by using BLAST v2.5.0.

The thorax of an individual healthy *T. sinensis* was used for library construction with Hi-C technology. A Hi-C library was constructed and sequenced on the Illumina platform, generating a total of 225.25 Gb raw data. To perform chromosome-level assembly of this genome based on chromatin conformation capture technology, the cleaned Hi-C read pairs were aligned to the assembled contigs using Juicer v1.6 (RRID:SCR_017226) [48], transforming raw data into a list of Hi-C contacts. Based on the alignment, the 3D-DNA v190716 (RRID:SCR_017227) pipeline [49] was applied to correct the initially assembled contigs with parameters “-r 2.” The 3D-DNA final assembly was adjusted using JuiceBox v1.11.08 (RRID:SCR_021172) [50], and then the scaffolds were further assembled into super-scaffolds.

### Annotation of repeats

The transposon was detected using EDTA v1.9.6 (RRID:SCR_022063) [51], and TRF v4.09 (RRID:SCR_022193) [52] was used to detect the tandem repeats in the *T. sinensis* genome, making a self-repeat database by the result of EDTA and TRF. A *de novo* repeat database was built using RepeatModeler v2.0.2 (RRID:SCR_015027) [53]. The known repeats in the Dfam database (RRID:SCR_021168) [54] and the self-repeat database were combined with all_rep_lib.fa, which was clustered by CD-HIT v4.8.1 (RRID:SCR_007105) [55] to remove redundant sequences. After combining and clustering, comprehensive repeat and TE detection was conducted using RepeatMasker v4.1.2 (RRID:SCR_012954) [56] with parameters “-lib all_rep_lib.fa.” In addition, the insertion time of each class of transposons was estimated by Kimura distance-based analysis [57] using parseRM [58].

### Transcriptome sequencing, protein-coding gene prediction, and annotation

After carefully removing the intestinal substances, PolyA (+) RNAs were extracted from an individual whole insect. The paired-end sequencing raw data generated by the Illumina NovaSeq 6000 sequencing platform were 16.62 Gb in fastq format, which would be used in the expression-based method for gene prediction. Fastp v0.20.1 (RRID:SCR_016962) [40] was used to trim the RNA sequencing raw reads for removing Illumina adapter sequences.

Transcriptome sequencing, homologous gene search, and *de novo* prediction were used to infer the protein-coding genes in the *T. sinensis* genome and integrated into a final gene set. First, the reads was aligned using Hisat2 v2.2.1 (RRID:SCR_015530) [59] and then assembled using StringTie v2.1.7 (RRID:SCR_016323) [60]. In parallel, the *de novo* assembly of the transcriptome sequence was conducted using Trinity v2.8.5 (RRID:SCR_013048) [61]. After combing the 2 assembly results, the transcriptome sequence was mapped to the genome for gene structural prediction using PASA v2.3.3 (RRID:SCR_014656) [62]. Second, homologous gene sets of manually annotated sequences from several kinds of insects in the Universal Protein Resource database (UniProt, RRID:SCR_002380) [63] and National Center for Biotechnology Information (NCBI) [64] were aligned to the *T. sinensis* genome sequence using Exonerate v2.4.0 (RRID:SCR_016088) [65] and Gemoma v1.7.1 (RRID:SCR_017646) [66]. Third, 3 programs, Augustus v3.3.3 (RRID:SCR_008417) [67], SNAP v2.54.3 (RRID:SCR_007936) [68], and GeneMark v4.65 (RRID:SCR_011930), were used for *de novo* gene prediction, respectively. The gene sets for Augustus and SNAP training were selected from the complete open reading frames prepared by PASA. Finally, all of the results were combined using EVidenceModeler v1.1.1 (RRID:SCR_014659) [69] into a nonredundant consensus of gene structures. To identify rRNAs, snRNAs, and miRNAs, we used Infernal 1.1.2 (RRID:SCR_011809) [70], which works by aligning sequences to the Rfam library [71].

To annotate the gene function, amino acid sequences of the predicted genes were aligned to the SwissProt, NT, and NR databases with the BLAST v2.5.0 [72] with an e-value threshold of 1e-5. Protein classification and domain search were achieved via the eggNOG-mapper v2.1.4 (RRID:SCR_021165) [73] and InterProScan v5.8.0 (RRID:SCR_005829) [74], and all implemented methods were utilized to assign Pfam domains, GO terms, and KEGG [75] pathway to gene models.

### Phylogeny and comparative genomics

*T. sinensis* genome and 13 other arthropod genomes with high-quality genomic assembly and publicly annotated gene information were selected from NCBI [64], i5k Workspace@NAL [76], and InsectBase [77] for comparative genomics analysis, including 6 Polyneoptera species (*Gryllus bimaculatus* [Orthoptera] [15], *Losta migratoria* [Orthoptera] [16, 17], *Clitarchus hookeri* [Phasmatodea] [18], *Blattella germanica* [Blattodea] [19], *Cryptotermes secundus* [Blattodea] [19], and *Zootermopsis nevadensis* [Blattodea] [20]) and 6 other insect species (*Ephemera danica* [Ephemeroptera] [21], *Rhodnius prolixus* [Hemiptera] [22], *Apis mellifera* [Hymenoptera] [23], *Drosophila melanogaster* [Diptera] [24], *Tribolium castaneum* [Coleoptera] [25], and *Bombyx mori* [Lepidoptera] [26]), and *Catajapyx aquilonaris* (Diplura: Japygidae) [27] was chosen as the outgroup. We used the longest transcript to represent the gene model when several transcripts of a gene were annotated. OrthoFinder v2.5.2 (RRID:SCR_017118) [78] was used to conduct homologous gene analysis for protein sequences of 14 insect genomes and search orthogroups. OrthoFinder with DIAMOND v2.0.5 (RRID:SCR_009457) [79] was used to align orthologroups with the parameter “-S diamond.” In addition, 221 conserved protein-coding genes were filtered from the orthogroups identified by OrthoFinder, occurring and presenting a single copy in all species, that were used to construct the phylogenetic tree. Multiple Alignment using Fast Fourier Transform (MAFFT) v7.480 (RRID:SCR_011811) [80] and FastTree (RRID:SCR_015501) [81] were used to cluster proteins into orthogroups, reconstruct gene trees, and estimate the species tree, and Alicut v2.31 [82] was used to cut randomized sequence sections in multiple sequence alignments in developing a super-sequence for each species. The OrthoFinder species tree was automatically rooted by OrthoFinder based on informative gene duplications. Further, ModelFinder in the IQ-Tree v2 package [83] could be used to predict the best model, and the phylogenetic tree was constructed based on Q.insect+R9 model by IQ-Tree v2 [84] with parameter “-bb 1000” using *C. aquilonaris* as the outgroup. The phylogenetic tree was visualized by FigTree v1.4.4 (RRID:SCR_008515) [85] and modified by iTOL (RRID:SCR_018174) [86].

MCMCTree in PAML v4.9j (RRID:SCR_014932) [87] was used to estimate species divergence time based on the Bayesian method. Since the sequence is an amino acid sequence, codeml in PAML should be used first when calculating the evolutionary rate using MCMCTree. A total of 7 reference divergence times were used as the calibration times: (i) Odonata, 221–235 Mya; (ii) Thysanoptera, 207–237 Mya; (iii) Hymenoptera, 211–235 Mya; (iv) Diptera, 94.3–99.7 Mya; (v) Coleoptera, 221–235 Mya; (vi) Holometabola/Hemiptera, 311.4–306.9 Mya; and (vii) fixed root, 479 Mya.

Following gene family clustering and divergence estimation, the expansion and contraction were analyzed using CAFÉ v4.2.1 (RRID:SCR_018924) [88] with the default parameters to calculate the probability of transition in each gene family from parent to child nodes in the phylogeny. The orthogroups information was obtained using orthofinder. For the CAFÉ analysis result, the gene families with family-wide *P* < 0.05 were defined as rapidly evolving families.

### Positive selection

The identification of positive selected sites in the phylogenetic tree was conducted by the branch model and branch site model with the Codeml tool of PAML v4.9j (RRID:SCR_014932) [87], respectively. A likelihood ratio test was performed to compare the fit of the 2 ratio models with the 1 ratio model to determine whether the gene was positively selected in the appointed branch (*P* < 0.05).

## Conclusions

The Chinese praying mantis is a natural predator insect that preys on various pests, making it a potential biological control agent. In our study, we used Illumina and PacBio sequencing with Hi-C scaffolding technology to generate the first chromosome-level genome assembly of Mantidae. Our findings reveal the significance of trypsin and GH gene expansions in prey digestion, as well as the importance of detoxification-related gene expansions, such as ABC transporter and CarE genes, in environmental adaptation. Furthermore, we identified 1 UV-sensitive opsin and 2 LWS opsins, emphasizing the crucial role of LWS opsins in modulating predatory behaviors. Our study not only offers a foundation for further applications of mantis in pest control but also sheds light on the genetic basis of mantis biology and evolution. Ultimately, our work serves as valuable biological information for researchers exploring the fascinating world of insect predators.

## Supplementary Material

giad090_GIGA-D-23-00141_Original_Submission

giad090_GIGA-D-23-00141_Revision_1

giad090_Response_to_Reviewer_Comments_Original_Submission

giad090_Reviewer_1_Report_Original_Submission Francesco Cicconardi -- 7/22/2023 Reviewed

giad090_Reviewer_2_Report_Original_Submission Reuben Nowell -- 7/26/2023 Reviewed

giad090_Supplemental_Files

## Data Availability

The *Tenodera sinensis* genome assembly is available in the NCBI database (GenBank accession JASJEM000000000). The raw sequencing data are available in the NCBI database under BioProject PRJNA971355. PacBio (SRR24501616) and Illumina (SRR24501617) sequencing data are available through the NCBI SRA. The Hi-C sequencing data are available from the BioProject page as NCBI accession SRR24501615. The paired-end Illumina RNA-seq data from an individual whole insect are available under NCBI SRR24501618. All additional supporting data are available in the *GigaScience* Database, GigaDB [89].

## Additional Files

**Supplementary Fig. S1**. Genomescope plot. The blue line means “observed,” the black line means “full model,” the yellow line means “unique sequence,” the red line means “errors,” and the dotted line means “*k*-mer peaks.”

**Supplementary Fig. S2**. Hi-C plot. The scale bar represents the interaction frequency of Hi-C links. Fourteen chromosomes were anchored in the genome, and the chromosome IDs were labeled aside each chromosome.

**Supplementary Fig. S3**. Histogram of hit reads of 2,189 sequences. The x-axis shows the species name hit to reads and the y-axis shows the number of hit reads. In the analysis, all of 2,189 sequences were conducted. Among them, there were 14 chromosome sequences and 2,175 debris reads.

**Supplementary Fig. S4**. Histogram of hit reads of 14 chromosomes. The x-axis shows the species name hit to reads and the y-axis shows the number of hit reads. In the analysis, 14 chromosome sequences were included, which make up 96.45% of the final assembly genome.

**Supplementary Fig. S5**. Distribution of LTR lengths. The x-axis shows the length of the LTR and the y-axis shows the frequency of occurrence.

**Supplementary Table S1**. The genome survey profile.

**Supplementary Table S2**. Repetitive sequences in the *Tenodera sinensis* genome assembly.

**Supplementary Table S3**. BUSCO summary in insecta_odb10.

**Supplementary Table S4**. BUSCO summary in arthropoda_odb10.

**Supplementary Table S5**. The statistics of GC content ratio.

**Supplementary Table S6**. The statistics of BLAST result in the Nr database.

**Supplementary Table S7**. The statistics of BLAST result in the Swiss-prot database.

**Supplementary Table S8**. The statistics of BLAST result in the TrEMBL database.

**Supplementary Table S9**. The statistics of Interproscan annotation result.

**Supplementary Table S10**. The statistics of EggNOG annotation result.

**Supplementary Table S11**. The statistics of ncRNA prediction.

**Supplementary Table S12**. The statistics of LTR landscape in *Tenodera sinensis*.

**Supplementary Table S13**. The statistics of LTR landscape in *Blattella germanica*.

**Supplementary Table S14**. The statistics of LTR landscape of *Cryptotermes secundus*.

**Supplementary Table S15**. The statistics of LTR landscape in *Zootermopsis nevadensis*.

**Supplementary Table S16**. The sequence of trypsin genes.

**Supplementary Table S17**. The sequence of glycosyl hydrolase genes.

**Supplementary Table S18**. The sequence of fatty acyl-coenzyme A reductase genes.

**Supplementary Table S19**. The sequence of ATP-binding cassette transporter genes.

**Supplementary Table S20**. The sequence of carboxylesterase genes.

**Supplementary Table S21**. The sequence of glutathione S-transferase genes.

**Supplementary Table S22**. The sequence of odorant receptor genes.

**Supplementary Table S23**. The sequence of ionotropic receptor genes.

**Supplementary Table S24**. The sequence of opsin genes.

## Abbreviations

ABC: ATP-binding cassette; BLAST: Basic Local Alignment Search Tool; BUSCO: Benchmarking Universal Single-Copy Orthologs; CarE: carboxylesterase; FacR: fatty acyl-coenzyme A reductase; GH: glycosyl hydrolase; GO: Gene Ontology; GST: glutathione S-transferase; Hi-C: high-throughput chromosome conformation capture; IR: ionotropic receptor; KEGG: Kyoto Encyclopedia of Genes and Genomes; lncRNA: long noncoding RNA; LWS: long-wavelength-sensitive; miRNA: microRNA; Mya: million years ago; NCBI: National Center for Biotechnology Information; ncRNA: noncoding RNAs; NGS: next-generation sequencing; OR: odorant receptor; rRNA: ribosomal RNA; SMRT: single-molecule real-time; snRNA: small nuclear RNA; sRNA: small RNA; TE: transposon element; tRNA: transfer RNA.
