# Supplementary material for: The chromosome-level genome of Chinese praying mantis Tenodera sinensis (Mantodea: Mantidae) reveals its biology as a predator
Source: Gigascience. 2023 Oct 26;12:giad090. doi: 10.1093/gigascience/giad090 (PMC10600911; doi:10.1093/gigascience/giad090)
Supplement: giad090_GIGA-D-23-00141_Revision_1 [file giad090_giga-d-23-00141_revision_1.pdf]

GigaScience

The chromosome-level genome of Chinese praying mantis Tenodera sinensis (Mantodea: Mantidae) reveals its biology as a predator

--Manuscript Draft--

**Manuscript Number:**	GIGA-D-23-00141R1
**Full Title:**	The chromosome-level genome of Chinese praying mantis Tenodera sinensis (Mantodea: Mantidae) reveals its biology as a predator
**Article Type:**	Data Note

Funding Information:

Funder	Recipient
the Key International Joint Research Program of National Natural Science Foundation of China (31920103005)	Dr Xuexin Chen
the General Program of National Natural Science Foundation of China (32070467)	Dr. Pu Tang
the Key Program of Regional Innovation and Development of National Natural Science Foundation of China (U22A20485)	Dr Xuexin Chen
the Provincial Key Research and Development Plan of Zhejiang (2021C02045)	Dr Xuexin Chen
the Fundamental Research Funds for the Central Universities (2021FZZX001-31)	Dr. Pu Tang

Abstract:

Background

The Chinese praying mantis, Tenodera sinensis (Saussure), is a beneficial insect that preys on various types of pests. Several studies have been conducted to understand its behavior and physiology. However, there is limited knowledge about the genetic information underlying its genome evolution, digestive demands, and predatory behaviors.

Findings

Here we have assembled the chromosome-level genome of T. sinensis, representing the first sequenced genome of the order Mantodea, with a genome size of 2.48 Gb and scaffold N50 of 174.78 Mb. Our analyses revealed that 96.4% of Benchmarking Universal Single-Copy Ortholog (BUSCO) genes are present, resulting in a well-annotated assembly compared to other insect genomes, containing 25,022 genes. We reconfirmed the phylogenetic position of the mantis within Insecta. Analysis of transposon elements suggested the Gypsy/Dirs family, which belongs to LTR transposons, may be a key factor resulting in the larger genome size. The genome shows expansions in several digestion and detoxification associated gene families, including trypsin and glycosyl hydrolase (GH) genes, ATP-binding cassette (ABC) transporter and carboxylesterase (CarE), reflecting the possible genomic basis of predatory digestive demands. Furthermore, we have found one ultraviolet-sensitive (UV-sensitive) opsin and two long-wavelength-sensitive (LWS) opsins, emphasizing the core role of LWS opsins in regulating predatory behaviors.

Conclusions

The high-quality genome assembly of the praying mantis provides a valuable repository for studying the evolutionary patterns of the mantis genomes and the gene expression profiles of insect predators.

**Corresponding Author:**	Pu Tang<br>Zhejiang University<br>Hangzhou, Zhejiang CHINA
**Corresponding Author Secondary**	

Powered by Editorial Manager® and ProduXion Manager® from Aries Systems Corporation

**Information:**	
**Corresponding Author's Institution:**	Zhejiang University
**Corresponding Author's Secondary Institution:**	
**First Author:**	Ruizhong Yuan
**First Author Secondary Information:**	
**Order of Authors:**	Ruizhong Yuan
	Boying Zheng
	Zekai Li
	Xingzhou Ma
	Xiaohan Shu
	Qiuyu Qu
	Xiqian Ye
	Sheng Li
	Pu Tang
	Xuexin Chen
**Order of Authors Secondary Information:**	
**Response to Reviewers:**	Reviewer #1: In this manuscript, Yuan and co-workers report the complete genome assembly of the Chinese praying mantis, Tenodera sinensis. The team used extensive genomic data to reconstruct the genome at the chromosome level, plus they perform a series of exploratory analyses to investigate genomic adaptation, such as gene expansions/contractions of the species.<br>How the new genomes were sequenced and annotated is sound and according to current standards in the field, making this work a useful resource for community. All the analyses were performed correctly, using correct methodologies. My only concern is related to the phylogenetic analyses, which is performed using a number of distant related species and only ~200 single-copy genes. It's a bit unfortunate because the approach is correct but the number of genes, used to build such a relatively deep phylogeny, is somehow small. I don't think that the small number of genes used may represent a problem for the topology, rather for the estimate of branch lengths and rates for the downstream analyses such as the dating. I'm personally not very surprised that orthofinder was not able to detect enough single-copy genes, but a better approach would be using BUSCO genes. In fact, the mantis genome has a high proportion of single-copy complete genes and the authors could leverage of the them to obtain a more robust set of data. I'm well aware that this would mean rerun some of the downstream analyses, therefore I leave of the authors/editor the decision on re-run the phylogeny.<br><br>Response: Due to the limited availability of genomic data for Mantodea and the scarcity of high-quality genomes in polyneoptera, our study focuses on reconstructing phylogenetic relationships on a broader scale. As a result, we obtained a relatively small number of single-copy genes from multiple insect genomes with distant relatives. Our phylogenetic tree constructed using the appropriate methodology aligns with the findings of certain previous studies (e.g., Misof et al., 2014). So we believe this topology can be utilized for further analysis, and therefore, we did not re-run the analysis at present.<br><br>Further comments:<br>Line 21: I'd remove "beneficial insect", not sure there's the need of anthropocentrism for the description of a new insect genome. I think it's valuable enough for the community on its own.<br><br>Response: Done. We have deleted the word “beneficial”.

Line 29-30: "We reconfirmed […] within Insecta." The authors should delete the sentence or rephrase is with something like "The reconstructed phylogenetic analysis show the expected topology placing …".

Response: Done. We have revised it.

Line 46: "A fascinating order of insects". I'm not aware of any non-fascinating order of insects.

Response: Done. We have deleted the word “fascinating”.

Line 69-70: "and genetic differences caused by …", I didn't quite understand what the authors mean with that.

Response: Done. What we mean is that there may be genetic diversity in the genomes of different geographic species

Line 86: de novo should be written in italics.

Response: Done.

Line 138: "Using orthofinder, we obtained a total of 216,767 genes".

Response: Done. We have revised it.

Line 141-143: The whole sentence is not clear, what are these 574 unique gene families? Maybe that there are 2436 genes with no orthology relationship clustered in 574 unique OGs?

Response: Done. We have revised it.

Line 153: I'd refer to orthologous groups rather than gene families. I think it's more appropriate.

Response: Done. We have revised it.

Line 188-190: Dating the estimation of effective population size has generally a lot of uncertainty on its own, I personally wouldn't be sure that the correlation to some human activity is also causal effect. I'd be very careful to jump to that conclusions. I'd remove the sentence.

Response: Done. We have deleted it.

Line 194: I'd remove "obviously".

Response: Done. We have deleted it.

Line 340-348: It's a bit confusing. Not sure what "transcriptome sequence was aligned" means in this context. Maybe the authors mean the reads? Also, there's a redundancy in the description of the de novo transcriptome assembly in the previous section (Line 340-342), repeated in the lines 347-348.

Response: Done. We have revised “transcriptome sequence” to “reads”, and deleted the redundancy description.

Line 386: I didn't understand the use of Diamond here. Can the authors clarify on that?

Response: DIAMOND is a sequence aligner for protein and translated DNA searches, designed for high performance analysis of big sequence data. Here, we used OrthoFinder to set the parameter “-S diamond” to execute the program.

Line 411-413: I'm not sure that the p value that CAFE returns is per se a sign of fast/rapid evolving OG, rather it should only be associated to the significance of that expansion/contraction statistical confidence.

Powered by Editorial Manager® and ProduXion Manager® from Aries Systems Corporation

Response: CAFÉ uses a statistical model to estimate the number of expansion and contraction events of a gene family at that node. The statistical significance of these changes was then assessed by comparing the observed changes in the number of families with the data from the random simulation and calculating a P-value. If the P-value of the change in the number of families at that node is less than a pre-set significance level (P-value < 0.05 were defined as rapidly evolving families in this study), then these gene families are considered to have evolved rapidly at that node.

Reviewer #2: Overall this is a well-presented genome note with another excellent addition to the growing genomic resource, this time for the praying mantis Tenodera sinensis. The underlying data and genome assembly/annotation methods presented are well explained and appear to be sound. The manuscript is generally well-written, but I found some of the conclusions made in the paper to be not particularly well explained in terms of how their data support the various hypotheses being presented. In addition, the Introduction uses quite a lot of "exceptional" language that should be moderated. Some care is needed to either (a) tone down the language or (b) present convincing evidence that support their ideas, or at least explain better how the present data do that. Nonetheless I am happy that the underlying data and genome assembly itself is of high quality and will no doubt be a useful genomic resource going forward.

Response: Many thanks for your positive comments. We have toned down the language in the text as suggested.

Specific comments:

Line 34: why these gene families specifically relating to "predatory digestive demands"

Response: Done. These gene families play a crucial role in the digestive process. Considering that the praying mantis primarily feeds on specific food sources, these gene families contribute significantly to the digestion of the prey within their digestive tracts. But we have removed the word “predatory” because of its inaccuracy and ambiguity.

Line 56: "predation character"—predatory nature?

Response: Done. We have revised it.

Throughout: use of exceptional terminology: "fascinating", "incredible", "unique" etc. E.g. line 48 "each boasting their own unique adaptations and behaviours"—surely this is true for any species?

Response: Done. We have thoroughly revised them in the text.

Line 62:

Response: Done.

Line 64: "fragmented studies"—do you mean studies using fragmented genome assemblies, or just that there have been only a few studies?

Response: Done. We mean the latter and have revised it.

Line 74: "valuable genomic resources for researchers studying predators"—this sounds too broad a claim, I'm not sure the mantis genome would tell us much about mammalian predators, for example… Do you perhaps mean "insect predators" specifically?

Response: Done. We have revised it.

Line 81: why would a "low level" of heterozygosity make it difficult to assemble? I would have thought it would be the other way around, unless you are referring to a fully

Powered by Editorial Manager® and ProduXion Manager® from Aries Systems Corporation

resolved (diploid) assembly.

Response: Done. We have revised it. Given the large size of the genome, assembling a chromosome-level genome may prove to be challenging.

Line 85: what is meant by "clean data"?

Response: Done. It means the data after quality control.

Line 97: the genome size quoted here (2.48 Gb) is different to that presented in Table 1 (2.54 Gb)?

Response: Done. We have revised it to the correct 2.54 Gb.

Line 99: please provide the total number of genes in the insecta and arthropoda BUSCO gene sets here or in Table 1.

Response: Done. We have provided it in Table 1.

Table 2: typo "Swiss-port" Swiss-prot

Response: Done.

Table 2: it is unclear what the numbers refer to in the "Functional annotation" column—are these numbers of genes with hits to the various databases? Please explain in the legend.

Response: Done. We have explained it in the legend.

Line 142: what is special about the 2436 genes here? Are these singleton genes unique to T. sinensis, whereas the 574 mentioned previously clustered into a gene family that was unique to T. sinensis?

Response: Done. It means there are 2436 genes with no orthologous relationship clustered in 574 unique OGs, and we have revised it.

Line 165: I'm a bit unconvinced by this. Of course these gene families may play a role in the mantis lifestyle, but is there any direct evidence for adaptive evolution here? It may just be a coincidence (i.e. drift), or driven by some other function unrelated to predation. I suggest toning down the strength of your remarks a little.

Response: Done. We have revised it.

Line 171: for the comparison of genome size, it would be useful to show the genome sizes of a few other species for comparison. ~2.5Gb seems very large for an insect, so it would be nice to see where this was on the distribution of genome sizes for insects/arthropods.

Response: We have considered this possibility and conducted relevant analyses. However, the presence of large genomes in primitive taxa, including Dictyoptera, poses challenges due to their low quality (evidenced by low BUSCO scores, low scaffold N50, and non-chromosome level genomes). The high computational requirements for analyzing these large genomes have also caused difficulties in our analysis. We hope that future research by other scientists can provide a more comprehensive explanation for the occurrence of large genomes in a broader range of insects/arthropods. We remain hopeful that our data can be valuable in the future when such explanations become available.

Line 180: genome duplication? Is the genome not diploid? Do you mean enlargement?

Response: Done. We have revised it.

Line 182: "significantly higher"—is there a statistical test to support this statement?

Powered by Editorial Manager® and ProduXion Manager® from Aries Systems Corporation

	Response: Done. In the sentence, “other insects” presents other three insects in Dictyoptera that we had analyzed in this part. The mantis showed 18.47% of LTR retrotransposons in the proportion, which is higher than other three insects in Dictyoptera (Figure 4. B). The expression used in the sentence was not precise, and we have made revision accordingly.<br><br>Line 194: delete "obviously", I'm not sure this is the correct meaning here.<br><br>Response: Done. We have deleted it.<br><br>Line 206: typo, delete "great"<br><br>Response: Done. We have deleted it.<br><br>Line 240: it's not clear how the location of these genes in the genome indicates their role in "intricate diet mechanisms" in this insect? Perhaps a bit more explanation needed.<br><br>Response: Done. What we are trying to explain here is that their role in the diet mechanism is represented by "multiple copies", not their location. Due to the average of our writing, which led to the ambiguity, we have revised it.<br><br>Line 273: more explanation is needed to explain how a lower rate of Ka/Ks would explain the "peak sensitivity in the 'green' region without other peaks detected in the spectrum".<br><br>Response: Done. This sentence is based on the conclusion of "the more LWS opsins were identified" in the previous paragraph, and has nothing to do with “the Ka/Ks of UV-sensitive opsin" in this paragraph, which is a mistake in our writing and has been corrected.<br><br>Line 278: was the sequenced individual a male or a female?<br><br>Response: Done. We have added the gender description in “Sampling”.<br><br>Line 305: what steps were taken to ensure the final assembly did not contain any sequences from non-target organisms deriving from contaminants/co-symbionts in the DNA libraries? I'm sure the data are clean but it would be good to indicate that this check has been done (e.g., BlobTools).<br><br>Response: Done. We have aligned the final assembly to the NT library and counted the result to ensure that the final assembly does not contain any sequences of non-target organisms from contaminants/symbionts in the DNA library.<br><br>Line 387: only 221 single-copy genes? this seems very small, any ideas why?<br><br>Response: Due to the limited availability of genomic data for Mantodea and the scarcity of high-quality genomes in polyneoptera, our study focuses on reconstructing phylogenetic relationships on a broader scale, encompassing Insecta as a whole. As a result, we have obtained a relatively small number of single-copy genes from multiple insect genomes with distant relatives. However, despite this limitation, our phylogenetic tree constructed using the appropriate methodology aligns with the findings of certain previous studies (e.g., Misof et al., 2014). We believe that this topology can be utilized for further analysis.<br><br>Line 391: typo "proteins"<br><br>Response: Done.<br><br>--
**Additional Information:**	
**Question**	**Response**

Powered by Editorial Manager® and ProduXion Manager® from Aries Systems Corporation

Are you submitting this manuscript to a special series or article collection?	No
**Experimental design and statistics**<br><br>Full details of the experimental design and statistical methods used should be given in the Methods section, as detailed in our Minimum Standards Reporting Checklist. Information essential to interpreting the data presented should be made available in the figure legends.<br><br>Have you included all the information requested in your manuscript?	Yes
**Resources**<br><br>A description of all resources used, including antibodies, cell lines, animals and software tools, with enough information to allow them to be uniquely identified, should be included in the Methods section. Authors are strongly encouraged to cite Research Resource Identifiers (RRIDs) for antibodies, model organisms and tools, where possible.<br><br>Have you included the information requested as detailed in our Minimum Standards Reporting Checklist?	Yes
**Availability of data and materials**<br><br>All datasets and code on which the conclusions of the paper rely must be either included in your submission or deposited in publicly available repositories (where available and ethically appropriate), referencing such data using a unique identifier in the references and in the "Availability of Data and Materials" section of your manuscript.<br><br>Have you have met the above requirement as detailed in our Minimum	Yes

Standards Reporting Checklist?	

Manuscript

Click here to access/download;Manuscript;Tsin - Manuscript_Oct 1.docx

The chromosome-level genome of Chinese praying mantis *Tenodera sinensis* (Mantodea: Mantidae) reveals its biology as a predator

Ruizhong Yuan[1, 2], Boying Zheng[1, 2], Zekai Li[1, 2], Xingzhou Ma[1, 2], Xiaohan Shu[1, 2, 3], Qiuyu Qu[1, 2, 3], Xiqian Ye[1, 2], Sheng Li[4, 5]*, Pu Tang[1, 2]*, Xuexin Chen[1, 2, 3‡]

1 Institute of insect Sciences, college of Agriculture and Biotechnology, Zhejiang University, Hangzhou, 310058, China.

2 State Key Lab of Rice Biology, Ministry of Agriculture Key Lab of Molecular Biology of crop Pathogens and insects, and Zhejiang Provincial Key Laboratory of Biology of crop Pathogens and Insects, Zhejiang University, Hangzhou, 310058, China.

3 Hainan Institute, Zhejiang University, Sanya, 572025, China.

4 Guangdong Provincial Key Laboratory of Insect Developmental Biology and Applied Technology, Institute of Insect Science and Technology, School of Life Sciences, South China Normal University, Guangzhou, China

5 Guangmeiyuan R&D Center, Guangdong Provincial Key Laboratory of Insect Developmental Biology and Applied Technology, South China Normal University, Meizhou 514779, China

* Corresponding author address: Sheng Li, lisheng@scnu.edu.cn; Pu Tang, ptang@zju.edu.cn

‡ Senior author address: Xuexin Chen, xxchen@zju.edu.cn

Ruizhong Yuan [0000-0003-4968-9702];

Boying Zheng [0000-0003-3101-6091];

Xiaohan Shu [0000-0002-2119-9667];

Sheng Li [0000-0002-4217-2367];

Pu Tang [0000-0003-2296-9544];

Xuexin Chen [0000-0002-9109-8853]

Abstract

Background: The Chinese praying mantis, *Tenodera sinensis* (Saussure), is a carnivorous insect that preys on a variety of arthropods and small vertebrates, including pest species. Several studies have been conducted to understand its behavior and physiology. However, there is limited knowledge about the genetic information underlying its genome evolution, digestive demands, and predatory behaviors.

Findings: Here we have assembled the chromosome-level genome of *T. sinensis,* representing the first sequenced genome of the family Mantidae, with a genome size of 2.54 Gb and scaffold N50 of 174.78 Mb. Our analyses revealed that 98.6 % of Benchmarking Universal Single-Copy Ortholog (BUSCO) genes are present, resulting in a well-annotated assembly compared to other insect genomes, containing 25,022 genes. The reconstructed phylogenetic analysis showed the expected topology placing the praying mantis in an appropriate position. Analysis of transposon elements suggested the Gypsy/Dirs family, which belongs to LTR transposons, may be a key factor resulting in the larger genome size. The genome shows expansions in several digestion and detoxification associated gene families, including trypsin and glycosyl hydrolase (GH) genes, ATP-binding cassette (ABC) transporter and carboxylesterase (CarE), reflecting the possible genomic basis of digestive demands. Furthermore, we have found one ultraviolet-sensitive (UV-

sensitive) opsin and two long-wavelength-sensitive (LWS) opsins, emphasizing the core role of LWS opsins in regulating predatory behaviors.

Conclusions: The high-quality genome assembly of the praying mantis provides a valuable repository for studying the evolutionary patterns of the mantis genomes and the gene expression profiles of insect predators.

Keywords

Mantodea, *Tenodera sinensis*, Chromosome, Insect genomics, Mantis, Digestive demand, Predation behavior

1 Introduction

Mantodea, an order of insects, belongs to the larger Polyneoptera group. This diverse group encompasses a wide array of species, including grasshoppers, crickets, and even cockroaches, each boasting their own adaptations and behaviors [1-3]. Mantodea currently comprises 29 extant families and three fossil families, with almost 3,000 known extant species [4]. All mantis species are predators and feed on other organisms from the moment they hatch and in populations where space is limited, cannibalism is common, with females even consuming males on occasion [5, 6]. Species in the order Mantodea, particularly those within the family Mantidae, are ambush predators [7].

Tenodera sinensis Saussure (Mantodea: Mantidae; NCBI:txid406589) (Figure 1), commonly known as the Chinese praying mantis, is widely distributed in China [8]. Due to its predatory

nature, it has great biological control potential, which makes it an important species for pest management. Unlike other feeding insects, the praying mantis requires the digestion of higher amounts of protein and the metabolism of various toxins from venomous prey [9, 10]. Moreover, the praying mantis has a highly specialized visual system that allows it to detect and track prey with incredible accuracy, its compound eyes have a wide field of view and can detect both color and motion [11-13]. Additionally, the praying mantis has an ability to perceive depth, which is essential for accurately striking and capturing prey [14]. However, despite the importance of the praying mantis in pest management, there is limited information on relevant gene families due to there have been only a few studies on its digestive demand and vision ability. Genetic deciphering of the praying mantis provides valuable data and clues for understanding the gene expression profiles of predators.

High-quality chromosome-level genomes of mantis are essential for understanding the biological information of insect predators. However, due to issues such as large genome size, and genetic diversity caused by different geographical populations, high-quality genomic data for Mantodea is currently unavailable. In this study, we assembled the genome of *T. sinensis* at the chromosomal-level by combining Illumina, PacBio (Single Molecule real-time Sequencing) and Hi-C (High-through chromosome conformation capture) sequencing. The assembly of the genome provides valuable genomic resources for researchers studying insect predators, aiding in the development of biological control strategies, population genetics, and evolutionary and phylogeny studies of insect genomes. Overall, the high-quality mantis genome assembly will undoubtedly have a significant impact on the field of entomology and related research areas.

2 Results

2.1 Genome assembly

The genome survey of *Tenodera sinensis* was initially found to have a low level of heterozygosity (0.47%) in a large 2.42 Gb genome (Supplementary fig. S1, Supplementary table S1). Given the large size of the genome, assembling a chromosome-level genome may prove to be challenging. To assemble a high-quality genome, a combination of PacBio long-read and Illumina short-read sequencing was used. The primary genome assembly was based on the data after quality control (467.29 Gb) generated by the PacBio Sequel I platform. Subsequently, the *de novo* assembly of the long-read data obtained from PacBio sequencing was polished and improved using the next generation sequence (NGS) data (23.16 Gb). The resulting reference genome assembly for *T. sinensis* has a total length of 2.45 Gb, comprising 1,763 scaffolds and 1,820 contigs with N50 lengths of 3.07 Mb and 3.05 Mb, respectively.

The reference assembly of *T. sinensis* was further improved using High-throughput chromosome conformation capture (Hi-C) analysis with 315.96 Gb Hi-C data. Using the contig interaction frequency calculated from the alignment of pairs with contigs, 96.45% of reference genome sequences were found to be successfully anchored in 14 pseudochromosome groups (Supplementary fig. S2). This completion marked the first ever chromosome-level genome of Mantidae, with a genome size of 2.54 Gb and a scaffold N50 length of 174.78 Mb (Table 1). The quality of the final genome was assessed using BUSCO v5.2.2 with the insect_obd10 database (Table 1, Supplementary table S3) and arthropoda_obd10 database (Supplementary table S4). The

analysis identified 98.6 % of highly conserved insect genes, indicating that the assembled *T. sinensis* genome is of high quality and can be used for further functional and comparative genomics studies (Figure 2). In order to interrogate the *T. sinensis* genome assembly, we aligned each read of the genome to the NT library using BLAST. Out of the 2189 reads from the final assembled genome, 1606 very short debris reads (73.77% of the reads) were not compared, but among the reads that were compared to the database, 90.90% were related to arthropods, and 92.45% were related to insects (Supplementary fig. S3). While the debris reads were numerous, they did not contribute significantly to the overall genome size. For the 14 chromosomes accounted for 96.45% of the size, we aligned 14 chromosomes to the NT library. Among them, 64.29% were related to *T. sinensis*, 21.43% to *Timema bartmani*, 7.14% to *Onthophagus taurus*, and 7.14% to *Timema tahoe*, suggesting that the final assembly does not contain any sequences of non-target organisms from contaminants/symbionts in the DNA library. (Supplementary fig. S4).

Species	*Tenodera sinensis*
Lineage	Mantodea: Mantidae
Genome level	Chromosome
Genome size (Mb)	2537.11
GC content (%)	37.62
Sequence number	2189
Contig N50 (Mb)	2.36
Scaffold N50 (Mb)	174.78
Maximum scaffold length (bp)	422258719
Minimum scaffold length (bp)	1000
Complete BUSCO Score - Insecta	C:98.6%[S:95.2%,D:3.4%],F:0.5%,M:0.9%,n:1367
Complete BUSCO Score - Arthropoda	C:99.0%[S:95.8%,D:3.2%],F:0.4%,M:0.6%,n:1013

Table 1: Statics for genome assembly of *Tenodera sinensis*. “n” means the number of gene in BUSCO gene set.

2.2 Genome annotation

A combination of de novo, transcriptome data and homology-based methods were used for predicting gene models. The genome was found to have a total of 1.78 Gb repetitive sequences, which accounted for 72.85 % of the genome. The GC content of the genomic contigs was 37.62% (Supplementary table S5). Among the repetitive sequences, DNA transposons and LINEs were found to be the most predominant, accounting for 36.63 % and 10.77 %, respectively. (Supplementary table S2).

Structural Annotation

Genes	25022
Mean gene length (bp)	34430
Repeat (%)	72.85
Non-coding RNAs	
rRNA	482
miRNA	139
snRNA	336
tRNA	80972
sRNA	2
lncRNA	2
Functional annotation	
Nr	10788
Swiss-prot	7851
TrEMBL	10715
Interproscan	15560
EggNOG	10523
Unannotated	34.69%
Total annotated	65.31%

Table 2: Genome annotation of *Tenodera sinensis*. The numbers in the “Functional annotation” column represent the count of genes that have been matched or have hits in different databases for functional annotation.

Gene functional annotation is helpful for understanding the complex relationship between internal

genes and external traits. The models of protein-coding genes were identified using de novo and homology-based prediction methods based on the transcriptome data, resulting in the identification of 25,022 protein-coding genes. Furthermore, functional annotation was performed to identify 19521 GO terms, 5363 KEGG ko terms, 2104 enzyme codes, 758 KEGG pathways and 3478 COG categories (Table 2, Supplementary table S6-10). Besides protein-coding genes, non-coding RNAs (ncRNAs) were also identified as important regulatory components in gene expression and epigenetics. Six types of ncRNAs were identified, including 482 rRNAs, 139 microRNAs (miRNAs), 336 small nuclear RNAs (snRNAs), 80972 transfer RNAs (tRNAs), 2 small RNAs (sRNAs) and 2 long non-coding RNAs (lncRNAs) (Table 2, Supplementary table S11).

2.3 Phylogenetic analysis

Identifying homologous relationships among sequences from different species is crucial for improving our understanding of evolution and biodiversity. In this regard, we compared the protein-coding genes of *T. sinensis* with those of 12 representatives, including six polyneopteran species (*Gryllus bimaculatus* [Orthoptera] [15], *Losta migratoria* [Orthoptera] [16, 17], *Clitarchus hookeri* [Phasmatodea] [18], *Blattella germanica* [Blattodea] [19], *Cryptotermes secundus* [Blattodea] [19] and *Zootermopsis nevadensis* [Blattodea] [20]), and six other insect species (*Ephemera danica* [Ephemeroptera] [21], *Rhodnius prolixus* [Hemiptera] [22], *Apis mellifera* [Hymenoptera] [23], *Drosophila melanogaster* [Diptera] [24], *Tribolium castaneum* [Coleoptera] [25] and *Bombyx mori* [Lepidoptera] [26]), with *Catajapyx aquilonaris* (Diplura: Japygidae) [27] being an outgroup. Using OrthoFinder, a total of 216,767 genes were analyzed among the 14

species, of which 177,216 were clustered into 16,153 orthogroups. We also analyzed the genes of single-copy and multi-copy orthologs, as well as unique genes and unassigned orthologous genes for each species. In the *T. sinensis* genome, there are 2,436 genes with no orthology relationship are clustered in 574 unique orthologous groups when compared to the other 13 species.

To gain an understanding of Mantodea genomic evolution, we reconstructed a phylogenomic tree of the 14 species base on 221 single-copy orthologs (1,548,781 amino acids) (Figure 3. A). The phylogenetic relationships of 14 insect species were well recovered, with all the nodes being strongly supported. Our results indicated that the ancestors of *T. sinensis* originated in the Jurassic period, around 167.19 million years ago.

To investigate the rapidly evolving orthologous groups in *T. sinensis*, we used orthologous group evolution analysis to uncover the changes that have occurred in certain orthologous groups over time. We found 979 orthologous groups had undergone expansions, while 1,723 orthologous groups had experienced contractions. Out of these, 31 orthologous groups (25 expansions and 6 contractions) were recognized as rapidly evolving orthogroups (Table 3). The significantly expanded orthologous groups were primarily associated with digestion (trypsin), detoxification (carboxylesterase, ABC transporter), glycometabolism (glycosyl hydrolase) and DNA transposition (DDE superfamily endonuclease, PiggyBac transposable element-derived protein). The significantly contracted orthologous groups mainly focus on chemoreception (odorant receptor, ionotropic glutamate receptor), and we unexpectedly found other digestive related orthologous groups (glutathione S-transferase, fatty acyl-coenzyme A reductase) were

significantly contracted in the *T. sinensis* genome.

The rapidly expanded orthologous groups were further confirmed to be involved in metabolic detoxification, digestion, and secondary metabolite synthesis, as shown in the GO and KEGG enrichments (Figure 3. B and 3. C). These results indicated that *T. sinensis* processes strong digestion and detoxification ability, which may enable it to effectively respond to toxic compounds present in its prey.

OG number	Evolving type	Annotation
OG0000003	Expansion	Trypsin
OG0000038	Expansion	Zinc finger C2H2-type protein
OG0000040	Expansion	DDE superfamily endonuclease
OG0000099	Expansion	PIF1-like helicase
OG0000126	Expansion	Hypothetical protein
OG0000138	Expansion	Zinc finger BED-type protein
OG0000212	Expansion	DDE superfamily endonuclease
OG0000244	Expansion	Glycosyl hydrolase
OG0000275	Expansion	PiggyBac transposable element-derived protein
OG0000296	Expansion	Endonuclease-reverse transcriptase
OG0000337	Expansion	Serpin
OG0000338	Expansion	PiggyBac transposable element-derived protein
OG0000364	Expansion	Carboxylesterase type B
OG0000369	Expansion	Serpin Kazal-type
OG0000505	Expansion	Reverse transcriptase
OG0000529	Expansion	Hypothetical protein
OG0000559	Expansion	CRAL-TRIO lipid binding domain
OG0000633	Expansion	Ankyrin repeats
OG0000709	Expansion	ABC transporter
OG0001080	Expansion	Ankyrin repeats
OG0001361	Expansion	Hypothetical protein

OG0001367	Expansion	Hypothetical protein
OG0003219	Expansion	Testicular haploid expressed repeat
OG0004380	Expansion	Hypothetical protein
OG0004408	Expansion	Hypothetical protein
OG0000022	Contraction	Odorant receptor
OG0000041	Contraction	Short chain dehydrogenase
OG0000044	Contraction	Glutathione S-transferase
OG0000047	Contraction	Ionotropic glutamate receptor
OG0000076	Contraction	Odorant receptor
OG0000095	Contraction	Fatty acyl-coenzyme A reductase

Table 3: Rapidly evolving gene families during the evolution of *Tenodera sinensis*.

2.4 Evolution of genome size

The expansion of DDE superfamily endonuclease and PiggyBac transposable element-derived protein indicates the high activity of transposons in the *T. sinensis* genome, which may lead to large-scale genome duplication in the ancestry of Mantodea (Table 3). Analysis of transposon element (TE) types and the TE insertion time in four species in Dictyoptera showed significant differences in TE content and concentration of TE insertion times at the last 5 million years ago in the Dictyoptera (Figure 4. A, Supplementary table S12-15). However, the total length of TEs in the *T. sinensis* genome is about 1.8 to 3.3 times more than others, and the proportion of LTR content (~18.47 %) is much higher, suggesting that the recent outbreak of LTRs may cause a large-scale enlargement in the evolutionary process of *T. sinensis*, and driven the enlargement of its genome

size (Figure 4. B). It is noteworthy that the proportion of LTR retrotransposons in the *T. sinensis* genome is higher than in other three insects in Dictyoptera (Figure 4. B). We observed that the Gypsy/Dirs family was the predominant LTR type in the *T. sinensis* genome, (Figure 4. C), and most LTR retrotransposons are short length (< 2000 bp) (Supplementary fig. S5). The Gypsy/Dirs family can duplicate themselves within the genome and insert into new locations, resulting in changes in genome structure and function [28-30], which may be a key factor contributing to the large genome size of *T. sinensis* and its evolution. Furthermore, demographic analysis shows that the effective population size of *T. sinensis* tends to have a large fluctuation (Figure 4. D), with a growth of population size about 100,000 years ago.

2.5 Characteristic digestive demand in insect predators

Mantodea, a member of Dictyoptera, is different from other feeding insects in its predatory characteristics, including high digestive demand and detoxification capabilities. It was observed in *T. sinensis* genome that trypsin and glycosyl hydrolase (GH) showed expansion, while fatty acyl-coenzyme A reductase (FacR) showed contraction, indicating that *T. sinensis* has a strong ability to digest and metabolize proteins, sugars and lipids (Supplementary table S16-18). Furthermore, expansion was observed in the ATP-binding cassette (ABC) transporter and carboxylesterase (CarE), while the glutathione S transferase (GST) showed contraction, which may indicate predatory insects rely heavily on the detoxification gene family (Supplementary table S19-21). To gain further insight into the digestive demand proteins and detoxification characteristics of predatory insects, a comparison was made between the protein families of *T. sinensis* and those of omnivorous cockroaches and scavenging termites in Dictyoptera.

The praying mantis primarily feeds on insects that are rich in protein, which explains the number of trypsin and GHs found in the mantis genome compared to other insects. We identified 107 trypsin coding genes and 11 GHs in the *T. sinensis* genome, the highest numbers among Dictyopteran species. However, while the amount of trypsin in mantis is only slightly more abundant than in cockroaches and termites, it suggests that different pancreatic proteins may perform the function of protein digestion in different feeding insects. Interestingly, even though the number of FacRs has expanded in the *T. sinensis* genome, it is still lower compared to the cockroach and termites, suggesting that during the predatory evolution of *T. sinensis*, multiple copies of trypsin genes and GHs are present in the genome due to the great demand for protein digestion. Nonetheless, it emphasizes that even though the demand for lipid digestion may be less noticeable, FacRs still performs an indispensable function of protein digestion in insect predators.

The detoxification gene family, such as P450, ATP-binding cassette (ABC) transporter gene family, and carboxylesterase, plays a crucial role in insect feeding, digestion and metabolism. The ABC transporter gene family, one of the largest protein families that exist at all stages of life, acts as major active transporters, hydrolyzing ATP to transport toxic metabolites across membranes [31]. Similarly, carboxylesterase (CarE), an essential metabolic detoxification enzyme, is mainly involved in the hydrolysis of compounds containing ester bonds inside an organism's body [32]. It can metabolize and degrade harmful substances, preventing them from reaching target sites [33, 34]. In the *T. sinensis* genome, we observed an expansion of ABC transporter and carboxylesterase genes. We annotated 77 ABC transporter genes and 45 carboxylesterase genes (Figure 5),

indicating that the praying mantis has a strong detoxification ability, while the toxins in the praying mantis mostly come from the external environmental toxins and the internal toxins present in the prey organisms.

Our phylogenetic analysis revealed a distinct expansion in the ABCG gene subfamily within the ABC transporter gene family (Figure 6. A), which is consistent with previous studies revealing that ABCG genes in arthropods play a crucial role in eliminating cholesterol from the body [35, 36]. It is possible that ABCG genes in the praying mantis also perform a similar function. To investigate this further, we plotted the locations of the ABC gene family on chromosomes and identified that the ABCG gene subfamily was mainly distributed across chromosome 1 (Chr1), chromosome 4 (Chr4), chromosome 5 (Chr5) and chromosome 8 (Chr8) (Figure 6. B). We also observed that the ABCG gene subfamily genes occurred in clusters on Chr1, Chr4, Chr5 and Chr8. Furthermore, both the *white* gene and *scarlet* gene, as members of the ABCG gene subfamily, were found in clusters on Chr8, and the *scarlet* gene was found in clusters on Chr1, suggesting that the ABCG gene subfamily may have produced multiple copies through gene replication events during evolution. The presence of multiple copies of the ABCG gene subfamily indicates that it performs an important function in the praying mantis, which is possibly related to the complex dietary mechanisms of insect predators, while *white* genes and *scarlet* genes could play a vital role in detoxification alongside the ABCG gene subfamily.

2.6 Chemoreception and vision genes involved in insect predatory behaviors

Chemoreception performs crucial functions in predatory behaviors. Insects rely on two major gene

families, odorant receptors (ORs) and ionotropic receptors (IRs), to mediate their sense of smell and taste. Our research has revealed a significant reduction in the number of ORs and IRs in the *T. sinensis* genome (Figure 5, Table 3, Supplementary table S22-23). This suggests that while olfaction plays an important role in locating prey habit [37], the predation behavior of mantis may not primarily rely on the regulation of the olfactory system. Instead, it may be more influenced by vision or other sensation mechanisms.

Throughout their evolution, most mantises have transitioned from active hunters to predators that ambush their prey. *T. sinensis*, for example, primarily waits for prey by hiding in the stems and leaves of low plants at dusk, relying on their visual ability to locate prey. Electroretinography studies have suggested that the praying mantis possesses a vision with major peak sensitivity to the "green" region of the spectrum [38]. We identified three opsins in the *T. sinensis* genome, including one ultraviolet-sensitive (UV-sensitive) opsin and two long-wavelength-sensitive (LWS) opsins (Supplementary table S24). The more LWS opsins were identified may explain why the praying mantis have major peak sensitivity in the “green” region in the spectrum, while also emphasizing the core role of LWS opsins in regulating the prey behavior of *T. sinensis*. Overall, these results suggest that the praying mantis possesses at least two kinds of opsins at least, enabling them to distinguish colors in nature. This ability may cause *T. sinensis* to prey on insects that have more varied appearances in terms of color [39], while also making it easier for their hunting behaviors to be affected by disruptive or warning coloration in longer wavelength light.

We combined the opsins of Polyneoptera with those of representative species in Holometabola to

test the phylogenetic relationship (Figure 6. C). Our analysis revealed that the UV-sensitive opsin of Polyneoptera formed a distinct clade, indicating the independent evolution of UV-sensitive opsin in this group. Subsequently, we analyzed the rate ratio (ω) of nonsynonymous to synonymous nucleotide substitution rate (Ka/Ks ratio) of each UV-sensitive opsin and found that the UV-sensitive opsin of *T. sinensis* generally had lower Ka/Ks ratios compared to those of species in Polyneoptera (Figure 6. D). It suggests that the UV-sensitive opsin in *T. sinensis* evolved at a slower rate compared to other polyneopteran species.

3 Materials and methods

3.1 Sampling

The line of *Tenodera sinensis* was supported by the Lisheng Zhang team of the Chinese Academy of Agricultural Science. The rearing temperature was set at 23.5 ℃, the humidity was set at 80 %, and fruit flies and mealworms were used to feed the cultivation for multiple generations. Morphological identification and mitochondrial genome information both showed that the species was *T. sinensis*. An adult female *T. sinensis* was used for the genome sequencing.

3.2 Genome sequencing and survey

The genomic DNA was extracted using the FastPure Cell/Tissue DNA Isolation Mini Kit (Vazyme Biotech Co.,Ltd, Nanjing, China) according to the manufacturer's instructions. Whole-genome shotgun sequencing was performed using the PacBio and Illumina NovaSeq 6000 se (RRID:SCR_016387) quencing platforms. A single-molecule real-time (SMRT) bell library was constructed and run on a SMRT cell in the PacBio Sequel I system (Pacific Biosciences), generating

a total of 467.29 Gb raw data. The sequencing depth from the Pacbio sequencing platform was about 94.49 ×. The paired-end sequencing raw data generated by Illumina sequencing platform was 23.16 Gb, and its sequencing depth was about 9.34 ×.

Illumina paired-end sequenced raw reads for the genomic survey were filtered using Fastp v0.20.1 (RRID:SCR_016962) [40] preprocessor (set to default parameter) to remove low-quality reads, adaptors, and reads containing poly-N. The size of *T. sinensis* genome was estimated by a k-mer analysis of the Illumina clean reads. The clean reads in the next-generation sequencing (NGS) paired-end libraries were subjected to 21-mer frequency distribution analysis as follows: Genome size = (K-mernum/main peak depth) × (1-Error rate). The k-mer profile was thus generated using Jellyfish v2.2.10 (RRID:SCR_005491) [41] to calculate the k-mer number and distribution, and the content of repeated sequences and the heterozygosity were calculated by GenomeScope v1.00 (RRID:SCR_017014) [42].

3.3 Genome assembly

The primary assembly of the clean subreads from the PacBio platform was carried out using nextDenovo v2.5.0 [43], and corrected using Canu v2.1.1 (RRID:SCR_015880) [44]. The Illumina data were further used to polish and improve the genome assembly using nextPolish v1.4.0 [45]. The haplotigs and contig overlaps in a de novo assembly were removed using purge dups v1.2.5 (RRID:SCR_021173) [46] based on read depth. The quality of the reference genome sequence was evaluated using BUSCO v5.2.2 (RRID:SCR_015008) [47]. Finally, we aligned the final assembled genome sequence to the NT library to interrogate the genome assembly by using BLAST v2.5.0.

The thorax of an individual healthy *T. sinensis* was used for library construction with Hi-C technology. A Hi-C library was constructed and sequenced on the Illumina platform, generating a total of 225.25 Gb raw data. To perform chromosome-level assembly of this genome based on chromatin conformation capture technology. The cleaned Hi-C read pairs were aligned to the assembled contigs using Juicer v1.6 (RRID:SCR_017226) [48], transforming raw data into a list of Hi-C contacts. Based on the alignment, the 3D-DNA v190716 (RRID:SCR_017227) pipeline [49] was applied to correct the initially assembled contigs with parameters "-r 2". The 3D-DNA final assembly was adjusted using JuiceBox v1.11.08 (RRID:SCR_021172) [50], then the scaffolds were further assembled into super-scaffolds.

3.4 Annotation of repeats

The transposon was detected using EDTA v1.9.6 (RRID:SCR_022063) [51], and TRF v4.09 (RRID:SCR_022193) [52] was used to detect the tandem repeats in the *T. sinensis* genome, making a self-repeat database by the result of EDTA and TRF. A de novo repeat database was built using RepeatModeler v2.0.2 (RRID:SCR_015027) [53]. The known repeats in Dfam database (RRID:SCR_021168) [54] and the self-repeat database were combined with being all_rep_lib.fa that was clustered by CD-HIT v4.8.1 (RRID:SCR_007105) [55] to remove redundant sequences. After combining and clustering, comprehensive repeat and TE detection was conducted using RepeatMasker v4.1.2 (RRID:SCR_012954) [56] with parameters "-lib all_rep_lib.fa". In addition, the insertion time of each class of transposons were estimated by Kimura distance-based analysis [57] using parseRM [58].

3.5 Transcriptome sequencing, protein-coding gene prediction and annotation

After carefully removing the intestinal substances, PolyA (+) RNAs were extracted from an individual whole insect. The paired-end sequencing raw data generated by the Illumina NovaSeq 6000 sequencing platform was 16.62 Gb in fastq format, which would be used in expression-based method for gene prediction. Fastp v0.20.1 (RRID:SCR_016962) [40] was used to trim the RNA-seq raw reads for removing Illumina adapter sequences.

Transcriptome sequencing, homologous gene search and de novo prediction were used to infer the protein-coding genes in the *T. sinensis* genome, and integrated into a final gene set. First, the reads was aligned using Hisat2 v2.2.1 (RRID:SCR_015530) [59], and then assembled using StringTie v2.1.7 (RRID:SCR_016323) [60]. In parallel, the de novo assembly of the transcriptome sequence was conducted using Trinity v2.8.5 (RRID:SCR_013048) [61]. After combing the two assembly results, the transcriptome sequence was mapped to the genome for gene structural prediction using PASA v2.3.3 (RRID:SCR_014656) [62]. Second, homologous gene sets of manually annotated sequences from several kinds of insects in the Universal Protein Resource database (UniProt, RRID:SCR_002380) [63] and National Center for Biotechnology Information (NCBI) [64] were aligned to the *T. sinensis* genome sequence using Exonerate v2.4.0 (RRID:SCR_016088) [65] and Gemoma v1.7.1 (RRID:SCR_017646) [66]. Third, three programs, Augustus v3.3.3 (RRID:SCR_008417) [67], SNAP v2.54.3 (RRID:SCR_007936) [68] and GeneMark v4.65 (RRID:SCR_011930), were used for de novo gene prediction, respectively. The gene sets for Augustus and SNAP training were selected from the complete open reading frames prepared by

PASA. Finally, all of the results were combined using EVidenceModeler v1.1.1 (RRID:SCR_014659) [69] into a nonredundant consensus of gene structures. To identify rRNAs, snRNAs, and miRNAs, we used Infernal 1.1.2 (RRID:SCR_011809) [70], which works by aligning sequences to the Rfam library [71].

To annotate the gene function, amino acid sequences of the predicted genes were aligned to the SwissProt, NT and NR database with the BLAST v2.5.0 [72] with an e-value threshold of 1e-5. Protein classification and domain search were achieved via the eggNOG-mapper v2.1.4 (RRID:SCR_021165) [73] and InterProScan v5.8.0 (RRID:SCR_005829) [74] with all implemented methods were utilized to assign Pfam domains, GO terms and KEGG [75] pathway to gene models.

3.6 Phylogeny and comparative genomics

T. sinensis genome and 13 other arthropod genomes with high-quality genomic assembly and publicly annotated gene information were selected from NCBI [64], i5k Workspace@NAL [76] and InsectBase [77] for comparative genomics analysis, including six Polyneoptera species (*Gryllus bimaculatus* [Orthoptera] [15], *Losta migratoria* [Orthoptera] [16, 17], *Clitarchus hookeri* [Phasmatodea] [18], *Blattella germanica* [Blattodea] [19], *Cryptotermes secundus* [Blattodea] [19] and *Zootermopsis nevadensis* [Blattodea] [20]), six other insect species (*Ephemera danica* [Ephemeroptera] [21], *Rhodnius prolixus* [Hemiptera] [22], *Apis mellifera* [Hymenoptera] [23], *Drosophila melanogaster* [Diptera] [24], *Tribolium castaneum* [Coleoptera] [25] and *Bombyx mori* [Lepidoptera] [26]), and *Catajapyx aquilonaris* (Diplura: Japygidae) [27]

was chosen as outgroup. We used the longest transcript to represent the gene model when several transcripts of a gene were annotated. OrthoFinder v2.5.2 (RRID:SCR_017118) [78] was used to conduct homologous gene analysis for protein sequences of 14 insect genomes, and search orthogroups. OrthoFinder with DIAMOND v2.0.5 (RRID:SCR_009457) [79] was used to align orthologroups with the parameter "-S diamond". In addition, 221 conserved protein-coding genes were filtered from the orthogroups identified by OrthoFinder, occurring, and presenting a single copy in all species, that were used to construct the phylogenetic tree. Multiple Alignment using Fast Fourier Transform (MAFFT) v7.480 (RRID:SCR_011811) [80] and FastTree (RRID:SCR_015501) [81] were used to cluster proteins into orthogroups, reconstruct gene trees and estimate the species tree, and Alicut v2.31 [82] was used to cut randomized sequence sections in multiple sequence alignments in developing a super-sequence for each species. The OrthoFinder species tree was automatically rooted by OrthoFinder based on informative gene duplications. Further, ModelFinder in the IQ-Tree v2 package [83] could be used to predict the best model, and the phylogenetic tree was constructed based on Q.insect+R9 model by IQ-Tree v2 [84] with parameter "-bb 1000" using *C. aquilonaris* as outgroup. The phylogenetic tree was visualized by FigTree v1.4.4 (RRID:SCR_008515) [85], and modified by iTOL (RRID:SCR_018174) [86].

MCMCTree in PAML v4.9j (RRID:SCR_014932) [87] was used to estimate species divergence time based on Bayesian method. Since the sequence is an amino acid sequence, codeml in PAML should be used first when calculating the evolutionary rate using MCMCTree. A total 7 reference divergence times were used as the calibration times: (1) Odonata: 221-235 Mya; (2) Thysanoptera:

207-237 Mya; (3) Hymenoptera: 211-235 Mya; (4) Diptera: 94.3-99.7 Mya; (5) Coleoptera: 221-235 Mya; (6) Holometabola/Hemiptera: 311.4-306.9 Mya; (7) Fixed root: 479 Mya.

Following gene family clustering and divergence estimation, the expansion and contraction were analyzed using CAFÉ v4.2.1 (RRID:SCR_018924) [88] with the default parameters to calculate the probability of transition in each gene family from parent to child nodes in the phylogeny. The Orthogroups information was obtained using orthofinder. For the CAFÉ analysis result, the gene families with family-wide P-value < 0.05 were defined as rapidly evolving families.

3.7 Positive selection

The identification of positive selected sites in the phylogenetic tree was conducted by the branch model and branch site model in the Codeml tool of the PAML v4.9j (RRID:SCR_014932) [87], respectively. A likelihood ratio test was performed to compare the fit of the two ratio models with the one ratio model to determine whether the gene was positively selected in the appointed branch ($P < 0.05$).

4 Conclusions

The Chinese praying mantis is a natural predator insect that preys on various pests, making it a potential biological control agent. In our study, we used Illumina and PacBio sequencing with Hi-C scaffolding technology to generate the first chromosome-level genome assembly of Mantidae. Our findings reveal the significance of trypsin and glycosyl hydrolase (GH) gene expansions in prey digestion, as well as the importance of detoxification-related gene expansions, such as ATP-

binding cassette (ABC) transporter and carboxylesterase (CarE) genes, in environmental adaptation. Furthermore, we identified one UV-sensitive opsin and two LWS opsins, emphasizing the crucial role of LWS opsins in modulating predatory behaviors. Our study not only offers a foundation for further applications of mantis in pest control but also sheds light on the genetic basis of mantis biology and evolution. Ultimately, our work serves as valuable biological information for researchers exploring the fascinating world of insect predators.

Data availability

The *Tenodera sinensis* genome assembly is available in the NCBI database (GenBank accession JASJEM000000000). The raw sequencing data is available in the NCBI database under BioProject PRJNA971355. PacBio (SRR24501616) and Illumina (SRR24501617) sequencing data are available through the NCBI SRA. The Hi-C sequencing data are available from the BioProject page as NCBI accession SRR24501615. The paired-end Illumina RNA-seq data from an individual whole insect are available under NCBI SRR24501618. All additional supporting data are available in the *GigaScience* Database, GigaDB [89]

Additional Files

Supplementary Fig. S1. Genomescope plot. The blue line means “observed”, the black line means “full model”, the yellow line means “unique sequence”, the red line means “errors” and the dotted line means “kmer-peaks”

Supplementary Fig. S2. Hi-C plot. The scale bar represents the interaction frequency of Hi-C links. 14 chromosomes were anchored in the genome, and the chromosome ID were labeled aside

each chromosome

Supplementary Fig. 3. Histogram of hit reads of 2189 sequences. The X-axis shows the species name hit to reads and the Y-axis shows the number of hit reads. In the analysis, all of 2189 sequences were conducted. Among them, there were 14 chromosome sequences and 2175 debris reads.

Supplementary Fig. 4. Histogram of hit reads of 14 chromosomes. The X-axis shows the species name hit to reads and the Y-axis shows the number of hit reads. In the analysis, 14 chromosome sequences were included, which make up 96.45% of the final assembly genome.

Supplementary Fig. 5. Distribution of LTR lengths. The X-axis shows the length of the LTR and the Y-axis shows the frequency of occurrence.

Supplementary Table S1. The genome survey profile

Supplementary Table S2. Repetitive sequences in the *Tenodera sinensis* genome assembly

Supplementary Table S3. BUSCO summary in insecta_odb10

Supplementary Table S4. BUSCO summary in arthropoda_odb10

Supplementary Table S5. The statistics of GC content ratio

Supplementary Table S6. The statistics of BLAST result in Nr database

Supplementary Table S7. The statistics of BLAST result in Swiss-prot database

Supplementary Table S8. The statistics of BLAST result in TrEMBL database

Supplementary Table S9. The statistics of Interproscan annotation result

Supplementary Table S10. The statistics of EggNOG annotation result

Supplementary Table S11. The statistics of ncRNA prediction

Supplementary Table S12. The statistics of LTR landscape in *Tenodera sinensis*

Supplementary Table S13. The statistics of LTR landscape in *Blattella germanica*

Supplementary Table S14. The statistics of LTR landscape of *Cryptotermes secundus*

Supplementary Table S15. The statistics of LTR landscape in *Zootermopsis nevadensis*

Supplementary Table S16. The sequence of trypsin genes

Supplementary Table S17. The sequence of glycosyl hydrolase genes

Supplementary Table S18. The sequence of fatty acyl-coenzyme A reductas genes

Supplementary Table S19. The sequence of ATP-binding cassette transporter genes

Supplementary Table S20. The sequence of carboxylesterase genes

Supplementary Table S21. The sequence of glutathione S transferase genes

Supplementary Table S22. The sequence of odorant receptor genes

Supplementary Table S23. The sequence of ionotropic receptor genes

Supplementary Table S24. The sequence of opsin genes

Competing interests

The authors declare that they have no competing interests.

Funding

This work was supported by the Key International Joint Research Program of National Natural Science Foundation of China (31920103005), the General Program of National Natural Science Foundation of China (32070467), the Key Program of Regional Innovation and Development of National Natural Science Foundation of China (U22A20485), the Provincial Key Research and Development Plan of Zhejiang (2021C02045) and the Fundamental Research Funds for the

Central Universities (2021FZZX001-31).

Authors' Contributions

Conceptualization and supervision, X.C., P.T. and S.L.; Software, R.Y., B.Z. and X.M.; Investigation, R.Y, Z.L., X.S., Q.Q. and X.Y.; Writing – Original Draft Preparation, R.Y.; Writing – Review & Editing, X.C., P.T. and R.Y.; Visualization, R.Y.; Funding Acquisition, X.C. and P.T.

Acknowledgement

The line of *Tenodera sinensis* was supported by the Lisheng Zhang team of the Chinese Academy of Agricultural Science.

References

1. Klass KD. The proventriculus of the Dicondylia, with comments on evolution and phylogeny in Dictyoptera and Odonata (Insecta). Zool Anz. 1998;237(1):15-42.
2. Klass KD. The pregenital abdomen of a mantid and a cockroach: musculature and nerve topography, with comparative remarks on other Neoptera (Insecta: Dictyoptera). Dtsch Entomol Z. 1999;46(1):3-42.
3. Grimaldi D. A fossil mantis (Insecta: Mantodea) in Cretaceous amber of New Jersey, with comments on the early history of the Dictyoptera. Am Mus Novit. 1997;3204:1-11.
4. Ehrmann R. Mantodea: Gottesanbeterinnen de Welt. Münster: Natur und Tier Verlag; 2002.
5. Prete FR, Wolfe MM. Religious supplicant, seductive cannibal, or reflex machine - in search of the praying-mantis. J Hist Biol. 1992;25(1):91-136.

6. Kadoi M, Morimoto K, Takami Y. Male mate choice in a sexually cannibalistic species: male escapes from hungry females in the praying mantid *Tenodera angustipennis*. J Ethol. 2017;35(2):177-85.

7. Svenson GJ, Whiting MF. Phylogeny of Mantodea based on molecular data: evolution of a charismatic predator. Syst Entomol. 2004;29(3):359-70.

8. Ge DY, Chen XS. Review of the genus *Palaeothespis* Tinkham (Mantodea : Thespidae), with description of one new species. Zootaxa. 2008;1716:53-8.

9. Mebs D, Yotsu-Yamashita M, Arakawa O. The praying mantis (Mantodea) as predator of the poisonous red-spotted newt *Notophthalmus viridescens* (Amphibia: Urodela: Salamandridae). Chemoecology. 2016;26(3):121-6.

10. Nyffeler M, Maxwell MR, Remsen JV. Bird predation by praying mantises: a global perspective. Wilson J Ornithol. 2017;129(2):331-44.

11. Nityananda V, Tarawneh G, Henriksen S, et al. A novel form of stereo vision in the praying mantis. Curr Biol. 2018;28(4):588-93.

12. Rossel S. Spatial vision in the praying-mantis - is distance implicated in size detection. J Comp Physiol A -Neuroethol Sens Neural Behav Physiol. 1991;169(1):101-8.

13. Mathis U, Eschbach S, Rossel S. Functional binocular vision is not dependent on visual experience in the praying-mantis. Vis Neurosci. 1992;9(2):199-203.

14. Nagata T, Koyanagi M, Tsukamoto H, et al. Depth perception from image defocus in a jumping spider. Science. 2012;335(6067):469-71.

15. Ylla G, Nakamura T, Itoh T, et al. Insights into the genomic evolution of insects from cricket genomes. Commun Biol. 2021;4:733.

16. Wang XH, Fang XD, Yang PC, et al. The locust genome provides insight into swarm formation and long-distance flight. Nat Commun. 2014;5:1-9.

17. Yang PC, Hou L, Wang XH, et al. Core transcriptional signatures of phase change in the migratory locust. Protein Cell. 2020;11(9):696-7.

18. Wu C, Twort VG, Crowhurst RN, et al. Assembling large genomes: analysis of the stick insect (*Clitarchus hookeri*) genome reveals a high repeat content and sex-biased genes associated with reproduction. BMC Genom. 2017;18(1):884.

19. Harrison MC, Jongepier E, Robertson HM, et al. Hemimetabolous genomes reveal molecular basis of termite eusociality. *Nat Ecol Evol*. 2018;2(3):557-66.

20. Terrapon N, Li C, Robertson HM, et al. Molecular traces of alternative social organization in a termite genome. Nat Commun. 2014;5:3636.

21. The i5k Initiative. Genome assembly Edan_2.0. 2017. https://www.ncbi.nlm.nih.gov/assembly/GCA_000507165.2/. Accessed 12 Dec 2017.

22. Mesquita RD, Vionette-Amaral RJ, Lowenberger C, et al. Genome of Rhodnius prolixus, an insect vector of Chagas disease, reveals unique adaptations to hematophagy and parasite infection. Proc Natl Acad Sci U S A. 2015;112(48):14936-41.

23. Wallberg A, Bunikis I, Pettersson OV, et al. A hybrid de novo genome assembly of the honeybee, *Apis mellifera*, with chromosome-length scaffolds. BMC Genom. 2019;20(1):275.

24. Adams MD, Celniker SE, Holt RA, et al. The genome sequence of *Drosophila melanogaster*. Science. 2000;287(5461):2185-95.

25. Kim HS, Murphy T, Xia J, et al. BeetleBase in 2010: revisions to provide comprehensive genomic information for Tribolium castaneum. Nucleic Acids Res. 2010;38(Database

issue):D437-42.

26. Lu F, Wei Z, Luo Y, et al. SilkDB 3.0: visualizing and exploring multiple levels of data for silkworm. Nucleic Acids Res. 2020;48(D1):D749-55.

27. Thomas GWC, Dohmen E, Hughes DST, et al. Gene content evolution in the arthropods. Genome Biol. 2020;21:15.

28. Grandbastien MA. LTR retrotransposons, handy hitchhikers of plant regulation and stress response. Biochim Biophys Acta-Gene Regul Mech. 2015;1849(4):403-16.

29. Kidwell MG, Lisch DR. Perspective: Transposable elements, parasitic DNA, and genome evolution. Evolution. 2001;55(1):1-24.

30. Wicker T, Sabot F, Hua-Van A, et al. A unified classification system for eukaryotic transposable elements. Nature Reviews Genetics. 2007;8(12):973-82.

31. Dermauw W, Van Leeuwen T. The ABC gene family in arthropods: Comparative genomics and role in insecticide transport and resistance. Insect Biochem Mol Biol. 2014;45:89-110.

32. Heikinheimo P, Goldman A, Jeffries C, et al. Of barn owls and bankers: a lush variety of alpha/beta hydrolases. Structure. 1999;7(6):R141-R6.

33. Kontogiannatos D, Michail X, Kourti A. Molecular characterization of an ecdysteroid inducible carboxylesterase with GQSCG motif in the corn borer, *Sesamia nonagrioides*. J Insect Physiol. 2011;57(7):1000-9.

34. Nardini L, Christian RN, Coetzer N, et al. Detoxification enzymes associated with insecticide resistance in laboratory strains of *Anopheles arabiensis* of different geographic origin. Parasites Vectors. 2012;5:113.

35. Schmitz G, Langmann T, Heimerl S. Role of ABCG1 and other ABCG family members in

lipid metabolism. J Lipid Res. 2001;42(10):1513-20.

36. Von Eckardstein A, Langer C, Engel T, et al. ATP binding cassette transporter ABCA1 modulates the secretion of apolipoprotein E from human monocyte-derived macrophages. Faseb J. 2001;15(9):1555-61.

37. Ezaki K, Yamashita T, Carle T, et al. Aldehyde-specific responses of olfactory sensory neurons in the praying mantis. Sci Rep. 2021;11(1):1856.

38. Sontag C. Spectral sensitivity studies on visual system of praying mantis, *Tenodera sinensis*. J Gen Physiol. 1971;57(1):93-112.

39. Fabricant SA and Herberstein ME. Hidden in plain orange: aposematic coloration is cryptic to a colorblind insect predator. Behav Ecol. 2015;26(1):38-44.

40. Chen SF, Zhou YQ, Chen YR, et al. fastp: an ultra-fast all-in-one FASTQ preprocessor. Bioinformatics. 2018;34(17):884-90.

41. Marcais G, Kingsford C. A fast, lock-free approach for efficient parallel counting of occurrences of k-mers. Bioinformatics. 2011;27(6):764-70.

42. Vurture GW, Sedlazeck FJ, Nattestad M, et al. GenomeScope: fast reference-free genome profiling from short reads. Bioinformatics. 2017;33(14):2202-4.

43. Hu J, Wang Z, Sun Z, et al. An efficient error correction and accurate assembly tool for noisy long reads. bioRxiv. 2023; doi:10.1101/2023.03.09.531669.

44. Koren S, Walenz BP, Berlin K, et al. Canu: scalable and accurate long-read assembly via adaptive k-mer weighting and repeat separation. Genome Res. 2017;27(5):722-36.

45. Hu J, Fan JP, Sun ZY, et al. NextPolish: a fast and efficient genome polishing tool for long-read assembly. Bioinformatics. 2020;36(7):2253-5.

46. Guan DF, McCarthy SA, Wood J, et al. Identifying and removing haplotypic duplication in primary genome assemblies. Bioinformatics. 2020;36(9):2896-8.

47. Simao FA, Waterhouse RM, Ioannidis P, et al. BUSCO: assessing genome assembly and annotation completeness with single-copy orthologs. Bioinformatics. 2015;31(19):3210-2.

48. Durand NC, Shamim MS, Machol I, et al. Juicer Provides a One-Click System for Analyzing Loop-Resolution Hi-C Experiments. Cell Syst. 2016;3(1):95-8.

49. Dudchenko O, Batra SS, Omer AD, et al. De novo assembly of the Aedes aegypti genome using Hi-C yields chromosome-length scaffolds. Science. 2017;356(6333):92-5.

50. Durand NC, Robinson JT, Shamim MS, et al. Juicebox provides a visualization system for Hi-C contact maps with unlimited zoom. Cell Syst. 2016;3(1):99-101.

51. Ou SJ, Su WJ, Liao Y, et al. Benchmarking transposable element annotation methods for creation of a streamlined, comprehensive pipeline. Genome Biol. 2019;20(1):275.

52. Benson G. Tandem repeats finder: a program to analyze DNA sequences. Nucleic Acids Res. 1999;27(2):573-80.

53. Flynn JM, Hubley R, Goubert C, et al. RepeatModeler2 for automated genomic discovery of transposable element families. Proc Natl Acad Sci U S A. 2020;117(17):9451-7.

54. Storer J, Hubley R, Rosen J, et al. The Dfam community resource of transposable element families, sequence models, and genome annotations. Mob DNA. 2021;12(1):2.

55. Li WZ, Godzik A. Cd-hit: a fast program for clustering and comparing large sets of protein or nucleotide sequences. Bioinformatics. 2006;22(13):1658-9.

56. Tempel S. Using and understanding RepeatMasker. Methods Mol Biol. 2012;859:29-51.

57. Chalopin D, Naville M, Plard F, et al. Comparative analysis of transposable elements

highlights mobilome diversity and evolution in vertebrates. Genome Biol Evol. 2015;7(2):567-80.

58. Kapusta A, Suh A, Feschotte C. Dynamics of genome size evolution in birds and mammals. Proc Natl Acad Sci U S A. 2017;114(8):E1460-E9.

59. Kim D, Paggi JM, Park C, et al. Graph-based genome alignment and genotyping with HISAT2 and HISAT-genotype. Nat Biotechnol. 2019;37(8):907-16.

60. Pertea M, Pertea GM, Antonescu CM, et al. StringTie enables improved reconstruction of a transcriptome from RNA-seq reads. Nat Biotechnol. 2015;33(3):290-97.

61. Haas BJ, Papanicolaou A, Yassour M, et al. De novo transcript sequence reconstruction from RNA-seq using the Trinity platform for reference generation and analysis. Nat Protoc. 2013;8(8):1494-512.

62. Haas BJ, Delcher AL, Mount SM, et al. Improving the Arabidopsis genome annotation using maximal transcript alignment assemblies. Nucleic Acids Res. 2003;31(19):5654-66.

63. Bateman A, Martin MJ, Orchard S, et al. UniProt: the Universal Protein Knowledgebase in 2023. Nucleic Acids Res. 2023;51(D1):D523-D31.

64. Benson DA, Karsch-Mizrachi I, Lipman DJ, et al. GenBank. Nucleic Acids Res. 2010;38:D46-D51.

65. Slater GS, Birney E. Automated generation of heuristics for biological sequence comparison. BMC Bioinform. 2005;6:31.

66. Keilwagen J, Wenk M, Erickson JL, et al. Using intron position conservation for homology-based gene prediction. Nucleic Acids Res. 2016;44(9):e89.

67. Stanke M, Waack S. Gene prediction with a hidden Markov model and a new intron

submodel. Bioinformatics. 2003;19:II215-II25.
68. Korf I. Gene finding in novel genomes. BMC Bioinform. 2004;5:59.
69. Haas BJ, Salzberg SL, Zhu W, et al. Automated eukaryotic gene structure annotation using EVidenceModeler and the program to assemble spliced alignments. Genome Biol. 2008;9(1):r7.
70. Nawrocki EP, Eddy SR. Infernal 1.1: 100-fold faster RNA homology searches. Bioinformatics. 2013;29(22):2933-5.
71. Griffiths-Jones S, Moxon S, Marshall M, et al. Rfam: annotating non-coding RNAs in complete genomes. Nucleic Acids Res. 2005;33:D121-D4.
72. Altschul SF, Gish W, Miller W, et al. Basic local alignment search tool. J Mol Biol. 1990;215(3):403-10.
73. Huerta-Cepas J, Forslund K, Coelho LP, et al. Fast genome-wide functional annotation through orthology assignment by eggNOG-Mapper. Mol Biol Evol. 2017;34(8):2115-22.
74. Zdobnov EM, Apweiler R. InterProScan - an integration platform for the signature-recognition methods in InterPro. Bioinformatics. 2001;17(9):847-8.
75. Kanehisa M, Goto S, Kawashima S, et al. The KEGG databases at GenomeNet. Nucleic Acids Res. 2002;30(1):42-6.
76. Poelchau M, Childers C, Moore G, et al. The i5k Workspace@NAL-enabling genomic data access, visualization and curation of arthropod genomes. Nucleic Acids Res. 2015;43(D1):D714-D9.
77. Mei Y, Jing D, Tang SY, et al. InsectBase 2.0: a comprehensive gene resource for insects. Nucleic Acids Res. 2022;50(D1):D1040-D5.

78. Emms DM, Kelly S. OrthoFinder: phylogenetic orthology inference for comparative genomics. Genome Biol. 2019;20(1):238.

79. Buchfink B, Xie C, Huson DH. Fast and sensitive protein alignment using DIAMOND. Nat Methods. 2015;12(1):59-60.

80. Katoh K, Misawa K, Kuma K, et al. MAFFT: a novel method for rapid multiple sequence alignment based on fast Fourier transform. Nucleic Acids Res. 2002;30(14):3059-66.

81. Price MN, Dehal PS, Arkin AP. FastTree: Computing Large Minimum Evolution Trees with Profiles instead of a Distance Matrix. Mol Biol Evol. 2009;26(7):1641-50.

82. Patrick K. Alicut (Version 2.31). https://github.com/PatrickKueck/AliCUT/. Accessed 22 Feb 2017.

83. Kalyaanamoorthy S, Minh BQ, Wong TKF, et al. ModelFinder: fast model selection for accurate phylogenetic estimates. Nat Methods. 2017;14(6):587-9.

84. Minh BQ, Schmidt HA, Chernomor O, et al. IQ-TREE 2: New models and efficient methods for phylogenetic inference in the genomic era. Mol Biol Evol. 2020;37(5):1530-4.

85. Figtree (2018). Figtree (Version 1.4.4). http://tree.bio.ed.ac.uk/software/Figtree/. Accessed 26 Nov 2018.

86. Letunic I, Bork P. Interactive Tree Of Life (iTOL) v5: an online tool for phylogenetic tree display and annotation. Nucleic Acids Res. 2021;49(W1):W293-W6.

87. Yang ZH. PAML: a program package for phylogenetic analysis by maximum likelihood. Comput Appl Biosci. 1997;13(5):555-6.

88. Han MV, Thomas GWC, Lugo-Martinez J, et al. Estimating gene gain and loss rates in the presence of error in genome assembly and annotation using CAFE 3. Mol Biol Evol.

2013;30(8):1987-97.

89. Yuan R, Zheng B, Li Z, et al. Supporting data for "The chromosome-level genome of Chinese praying mantis *Tenodera sinensis* (Mantodea: Mantidae) reveals its biology as a predator" GigaScience Database. 2023. http://dx.doi.org/10.5524/102456.

PICTURE LEGENDS

Figure 1: The Chinese praying mantis (*Tenodera sinensis*) whose genome was sequenced, at Institute of Insect Sciences, Zhejiang University (Hangzhou, China). Photo by Xiqian Ye.

Figure 2: Genome assemble of *Tenodera sinensis*. “a” means chromosome ID, “b” means GC content, “c” means repetitive sequence content, and “d” means gene content.

Figure 3: Phylogenetic analyses of *Tenodera sinensis* and GO, KEGG of rapid evolved genes in expansion. A. Phylogenetic tree of *T. sinensis* and other 13 species. The estimated species divergence times (Million Years Age, MYA) are indicated at each branch point. Node values indicate gene families showing expansion (green) and contraction (blue). The bar chart indicates the number of genes classified into 5 groups (single-copy genes, multiple-copy genes, unique genes, other genes and unclustered genes). B. The GO enrichment of rapid evolved genes in expansion. C. The KEGG pathway analyses of rapid evolved genes in expansion.

Figure 4: Genome size evolution. A. The phylogenetic relationship between 4 species from Dictyoptera. B. TE insertion time and TE content. The bar chart shows the TE insertion time and its length (bp), and the pie chart shows the percentage of different TE types. C. The distribution of LTR type in *T. sinensis*. D. The demographic history of *T. sinensis*. The red line represents the pairwise sequentially Markovian coalesent (PSMC) estimate. The plot was constructed assuming a generation time of 1.00 years and mutation rate of 0.2×10^{-9} per generation.

Figure 5: Expansion and contraction of digestion, detoxification, and chemoreception gene families in

Dictyoptera. The size of circle means gene count of each gene families, and 4 species in Dictyoptera are counted, *T. sinensis* (red), *B. germanica* (orange), *C. secundus* (slight blue), *Z. nevadensis* (blue).

Figure 6: Analysis of ABC transport genes and visual genes. A. Expansion of the ABC transport gene family in *Tenodera sinensis*. The phylogenetic tree shows the orthologous and paralogous relationship of all 259 ABC transport genes from *T. sinensis* and other 3 species in Dictyoptera. 7 clades are marked as subfamilies, and gene labels of *T. sinensis* are marked in red. Bootstrap values are indicated on the node. B. The distribution of the ABC transport genes in *T. sinensis*. The ABCG subfamily genes are marked in orange. C. The phylogenetic tree of visual genes from *T. sinensis* and other 13 species. 3 clades are marked as long wavelength-sensitive (green), ultraviolet-sensitive (purple) and blue-sensitive (blue). The dN/dS (ω) values are indicated in the visual genes in *T. sinensis*. D. The dN/dS (ω) values of 6 species from Polyneoptera and other 4 species.

Figure 1 Click here to access/download;Figure;Figure 1.pdf

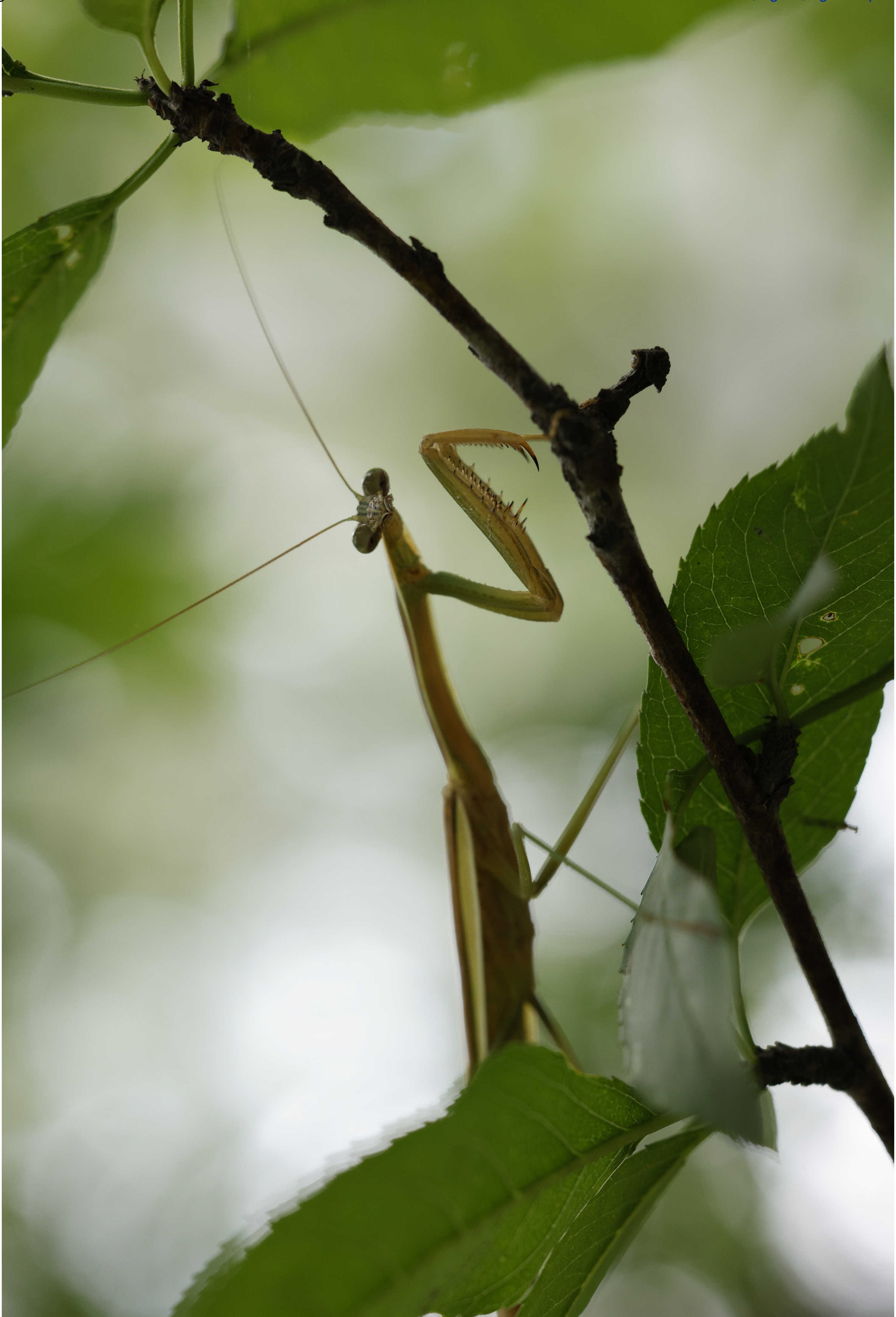

Figure 2

Click here to access/download;Figure;Figure 2.pdf

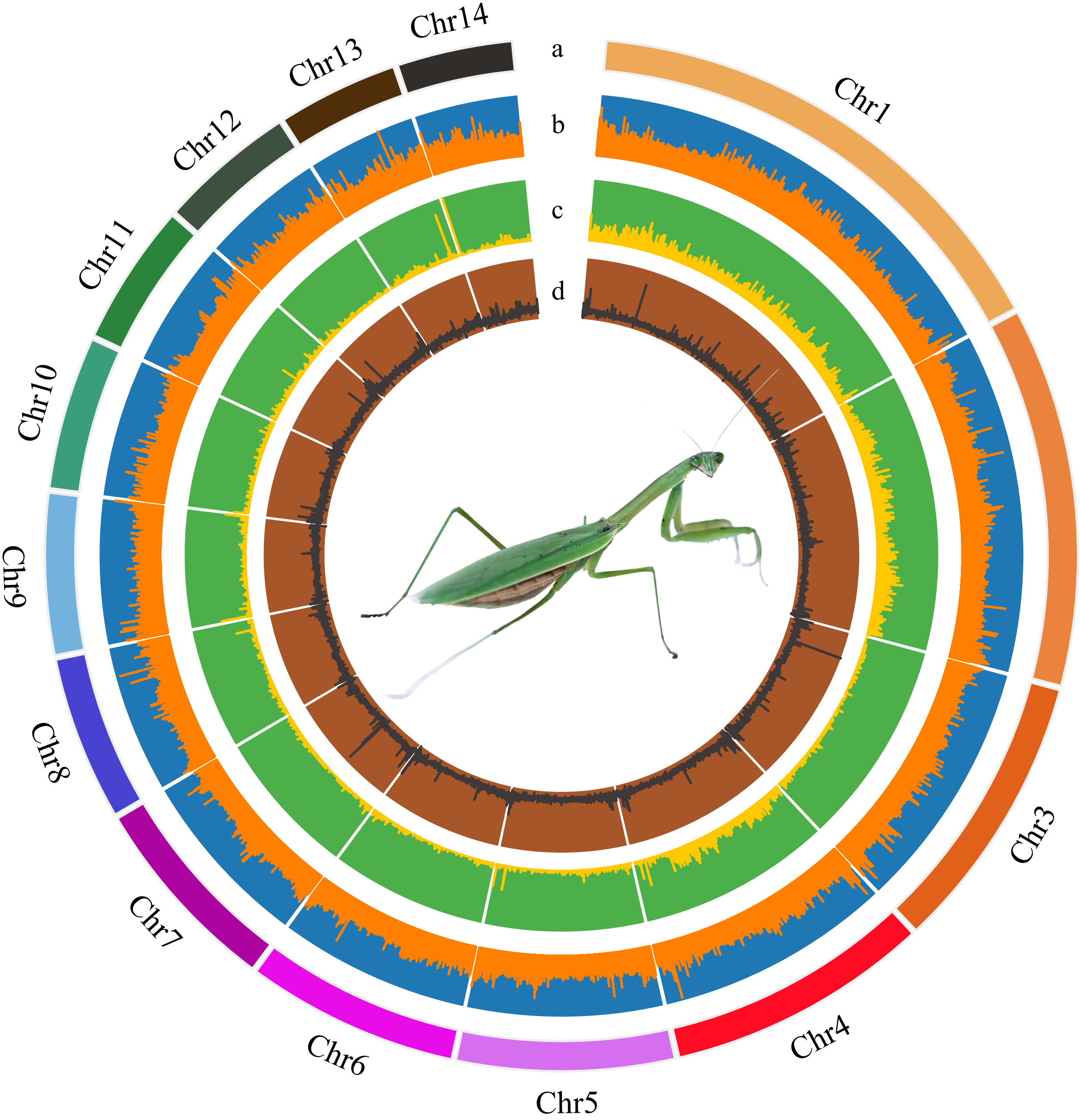

Figure 3

Click here to access/download;Figure;Figure 3.pdf

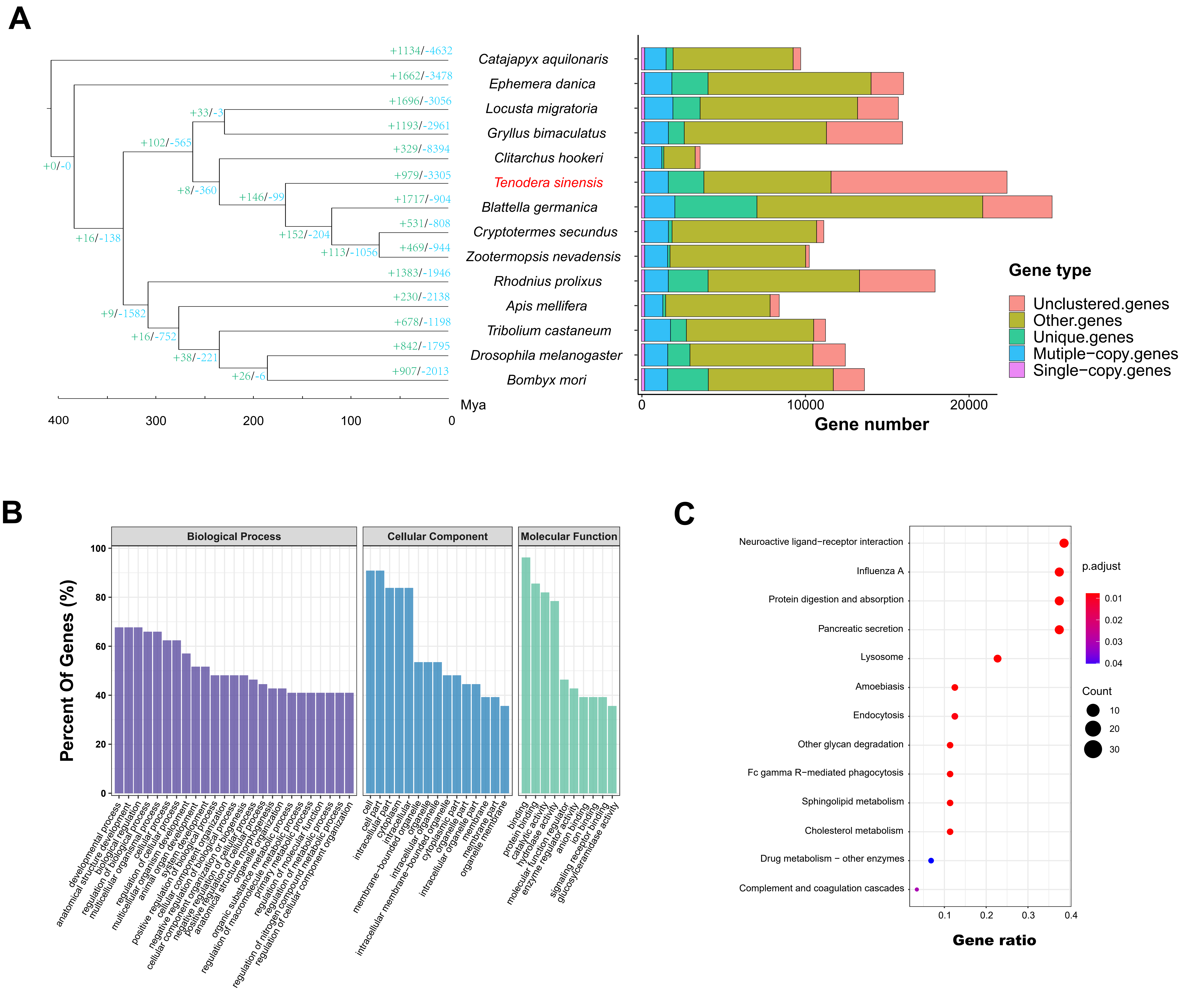

Figure 4

Click here to access/download;Figure;Figure 4.pdf

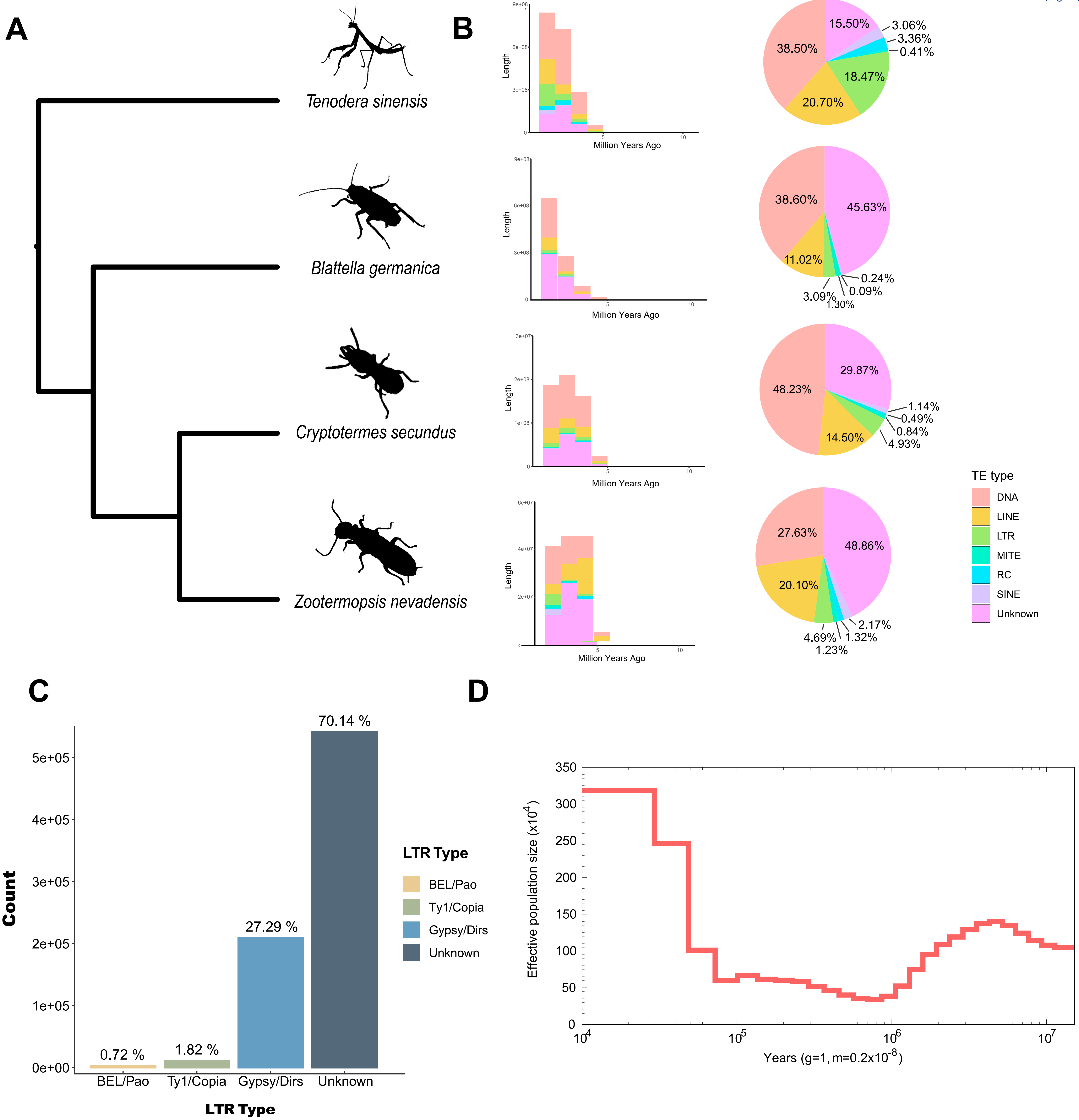

Figure 5

Click here to access/download;Figure;Figure 5.pdf

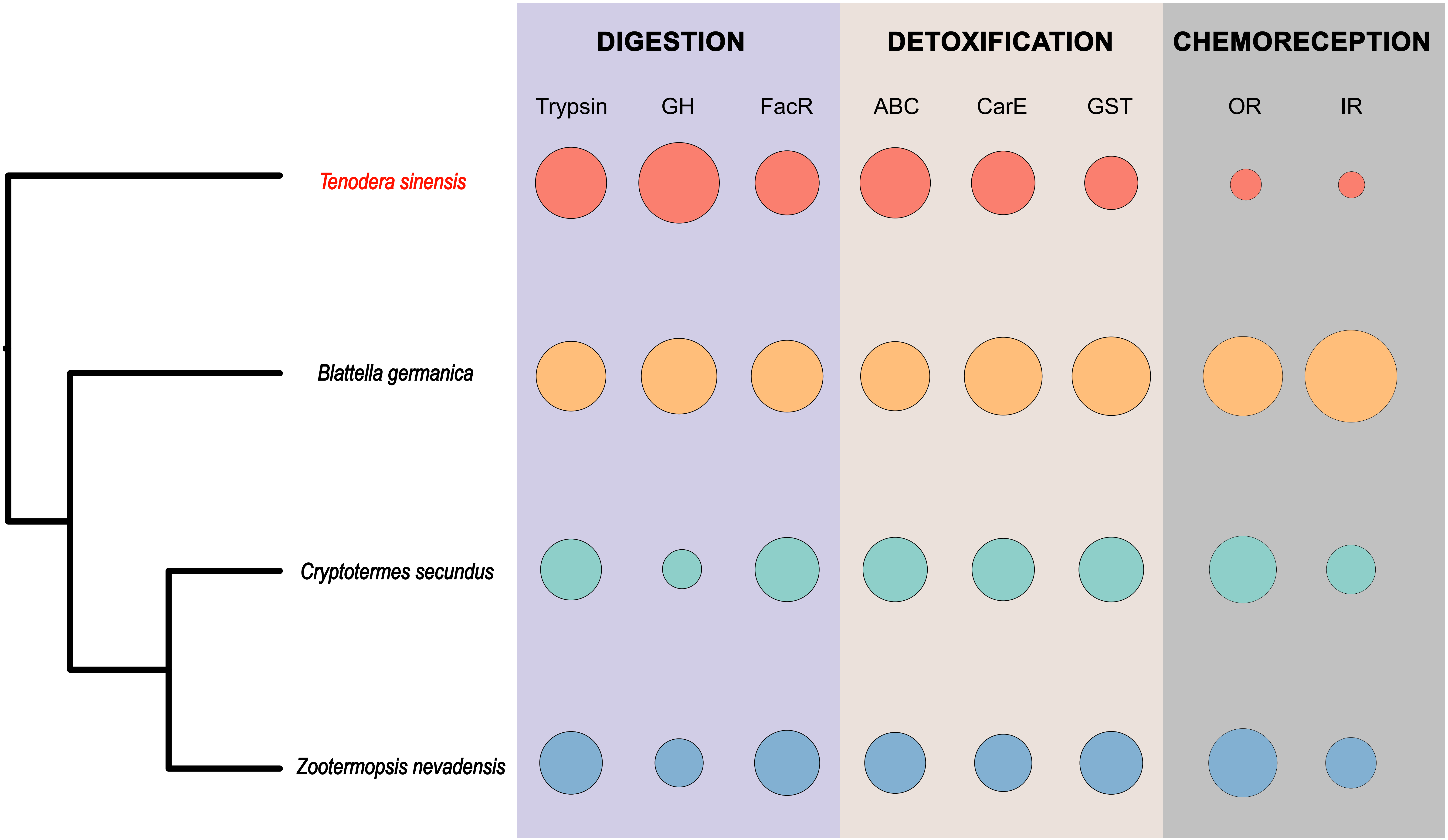

Figure 6

Click here to access/download;Figure;Figure 6.pdf

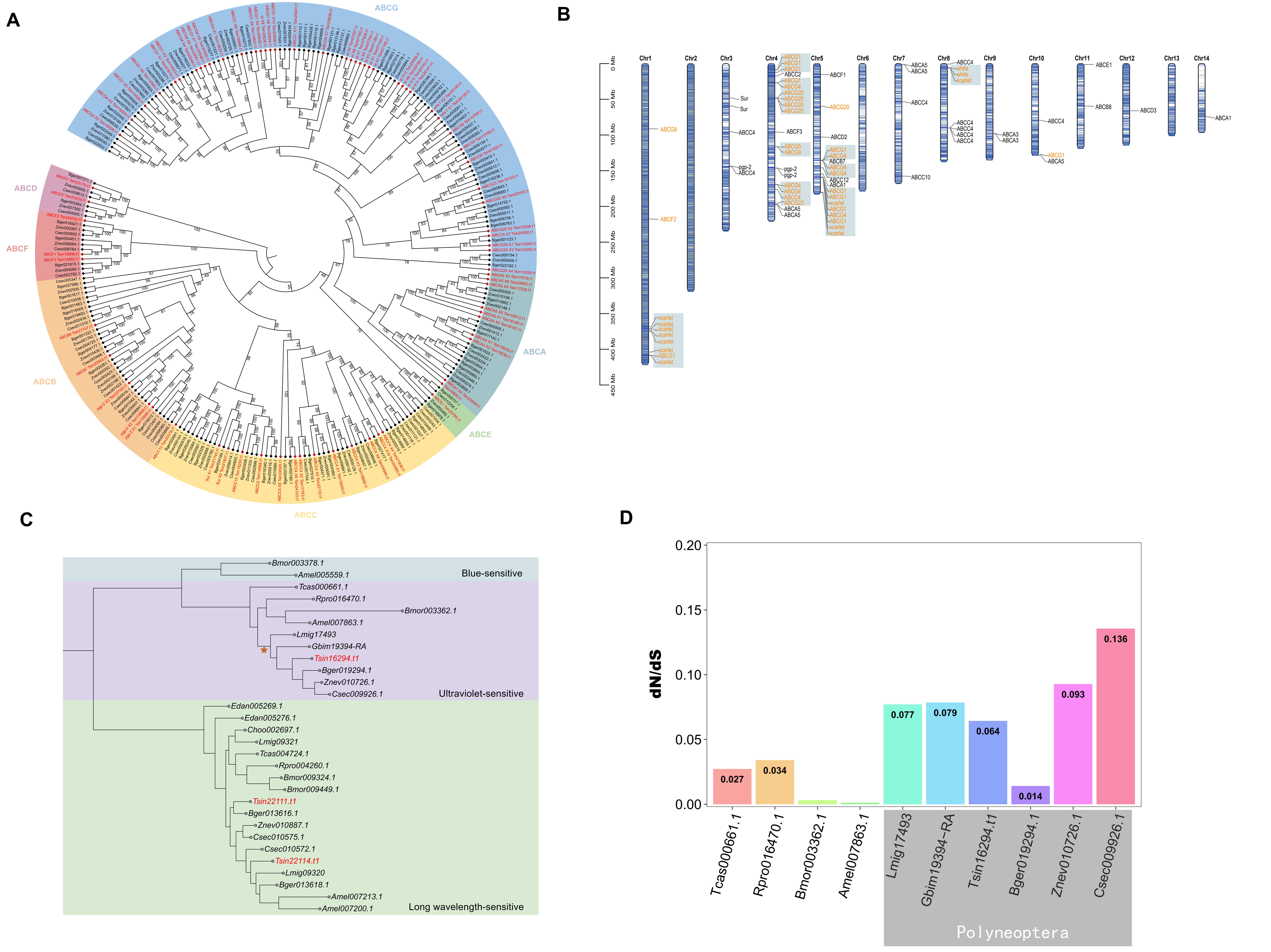

Click here to access/download
Supplementary Material
Supplementary Material_Tables.xlsx

Supplementary Fig. S1

Click here to access/download
Supplementary Material
Supplementary Material_Fig. S1.pdf

Supplementary Fig. S2

Click here to access/download
Supplementary Material
Supplementary Material_Fig. S2.pdf

Supplementary Fig. S3

Click here to access/download

Supplementary Material

Supplementary Material_Fig. S3.pdf

Supplementary Fig. S4

Click here to access/download
Supplementary Material
Supplementary Material_Fig. S4.pdf

Supplementary Fig. S5

Click here to access/download
Supplementary Material
Supplementary Material_Fig. S5.pdf

Personal Cover
Click here to access/download;Personal Cover;Tsin - Personal_Cover.docx

Sept. 17, 2023

The Editorial Office, *Gigascience.*

Dear Editors,

On behalf of all authors, we submitted a revision of our manuscript entitled "**The chromosome-level genome of Chinese praying mantis *Tenodera sinensis* (Mantodea: Mantidae) reveals its biology as a predator**".

We appreciate the careful review and constructive comments on our previous manuscript and we revised the paper according to the comments and suggestions of reviewers. We hope this revision addresses all the questions and comments, and hope it is now up to *Gigascience* standards for publication.

Sincerely,

Tang Pu
Professor of Entomology
Zhejiang University
