# Supplementary figures and images for: The chromosome-level genome of Chinese praying mantis Tenodera sinensis (Mantodea: Mantidae) reveals its biology as a predator
Source: Gigascience. 2023 Oct 26;12:giad090. doi: 10.1093/gigascience/giad090 (PMC10600911; doi:10.1093/gigascience/giad090)

# GenomeScope Profile

len:4,211,625,669bp uniq:61.8% het:0.468% kcov:19.9 err:0.345% dup:1.28% k:21

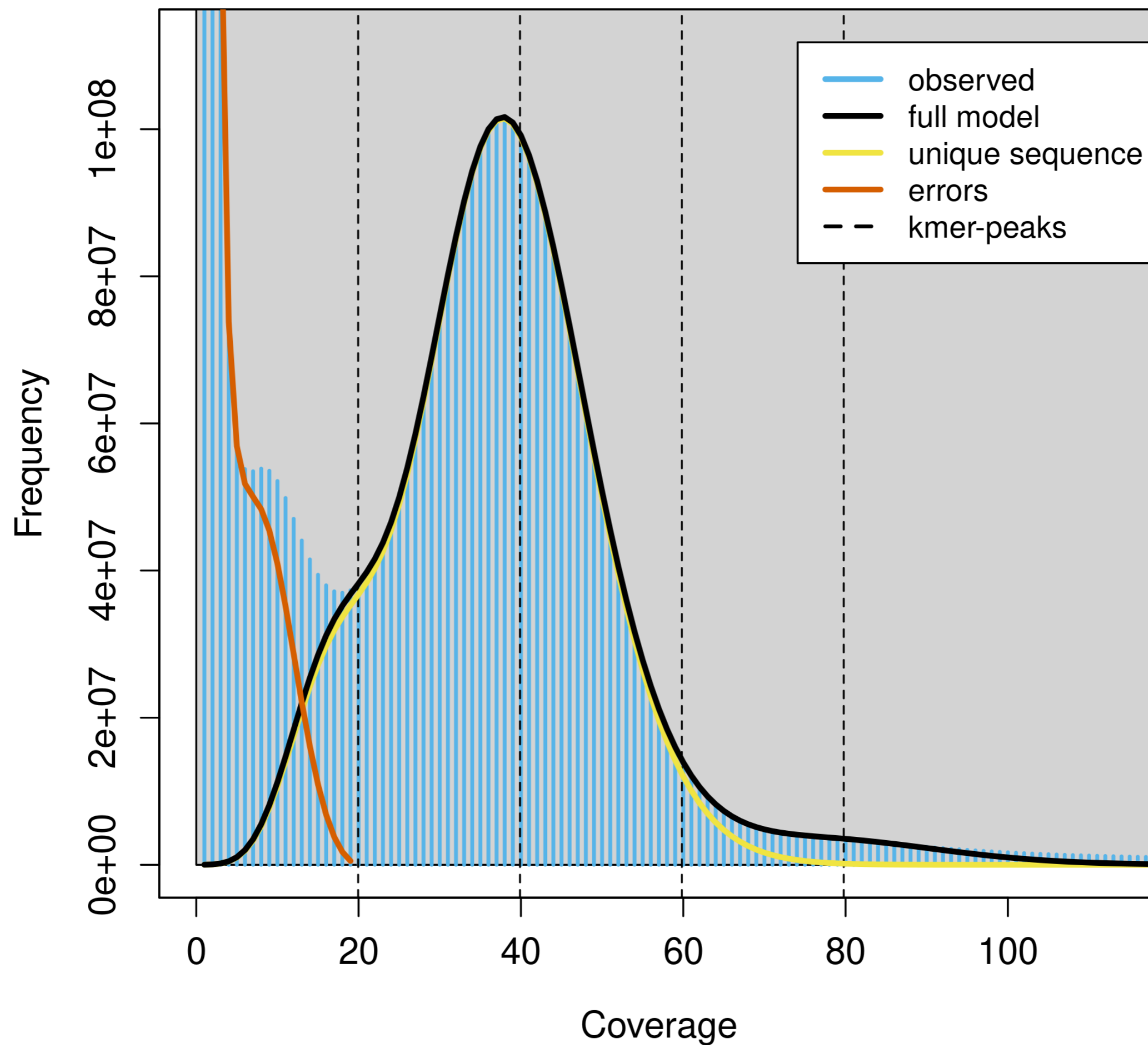

Supplement: giad090_Supplemental_Files [file giad090_supplemental_files.zip › Supplementary Material_Fig. S1.pdf]

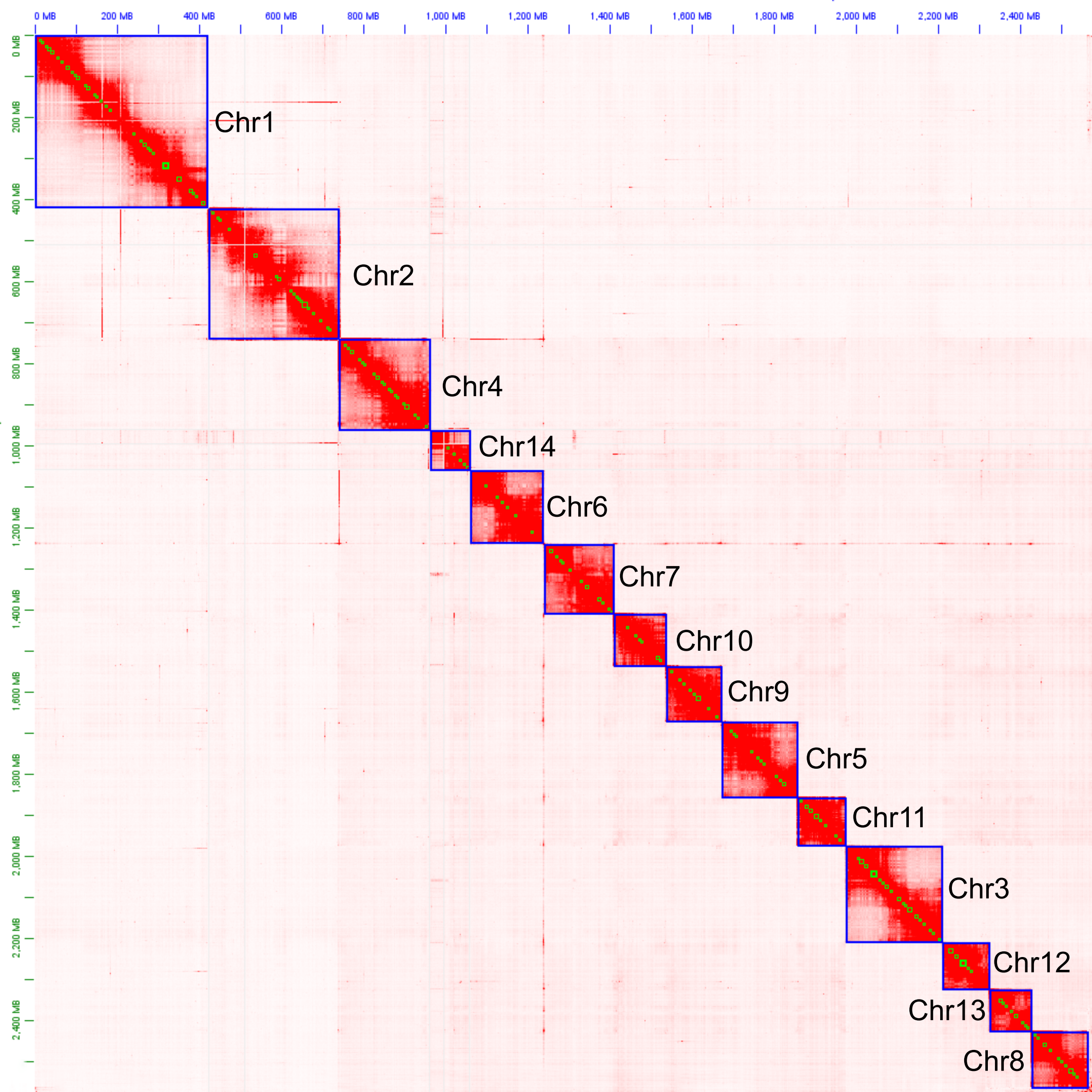

Supplement: giad090_Supplemental_Files [file giad090_supplemental_files.zip › Supplementary Material_Fig. S2.pdf]

Histogram of hit reads of 2189 sequences

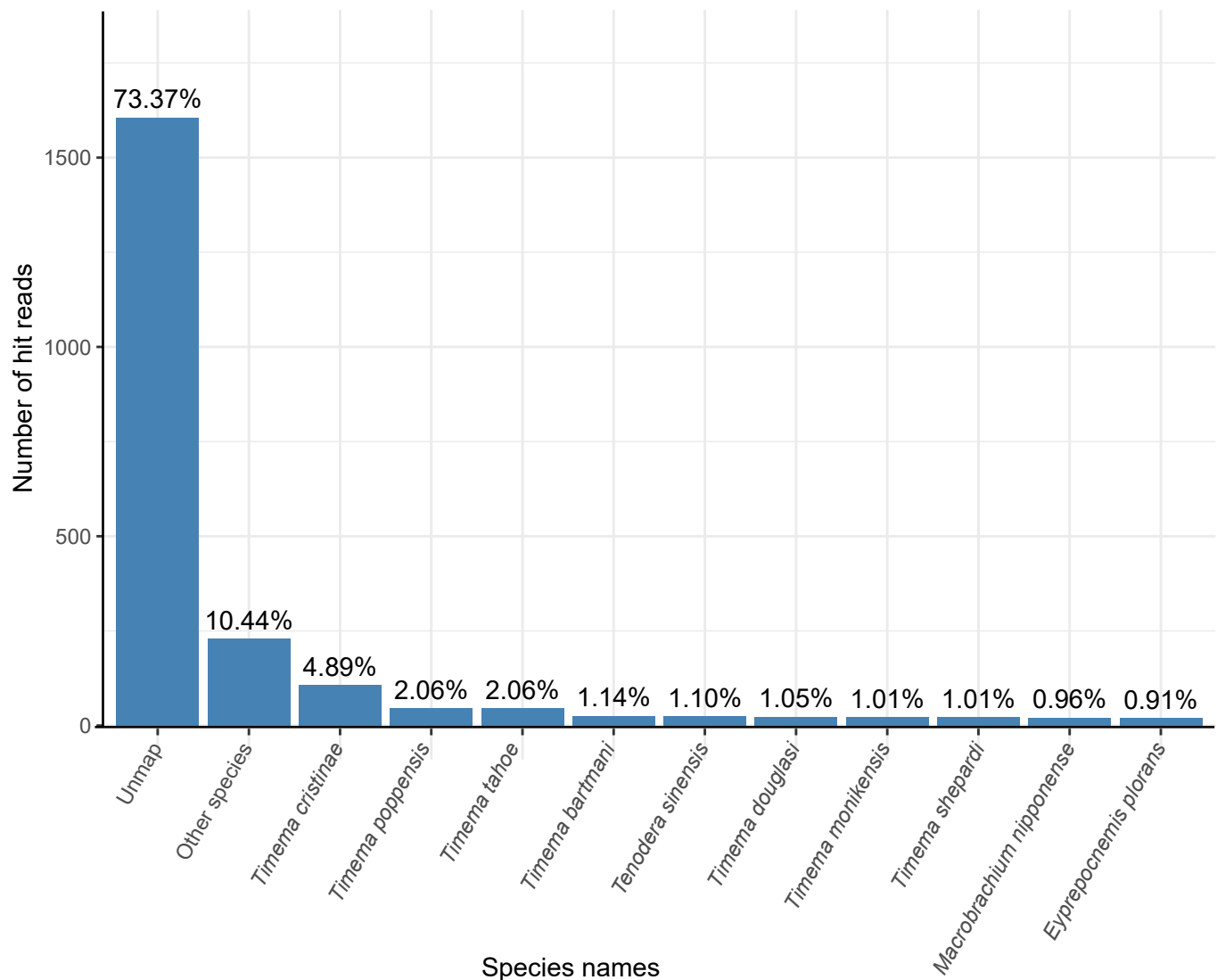

Supplement: giad090_Supplemental_Files [file giad090_supplemental_files.zip › Supplementary Material_Fig. S3.pdf]

Histogram of hit reads of 14 chromosomes

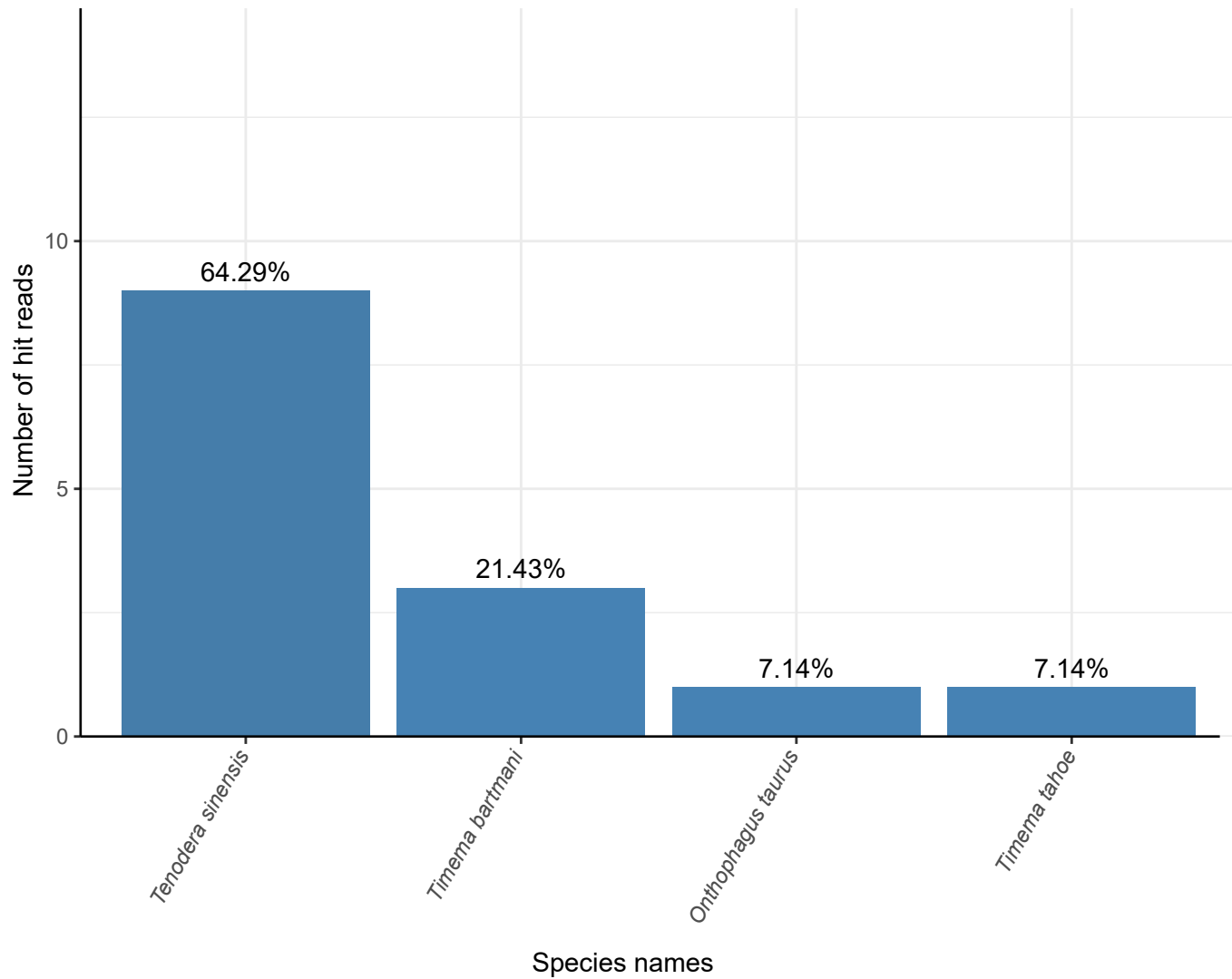

Supplement: giad090_Supplemental_Files [file giad090_supplemental_files.zip › Supplementary Material_Fig. S4.pdf]

Distribution of LTR Lengths

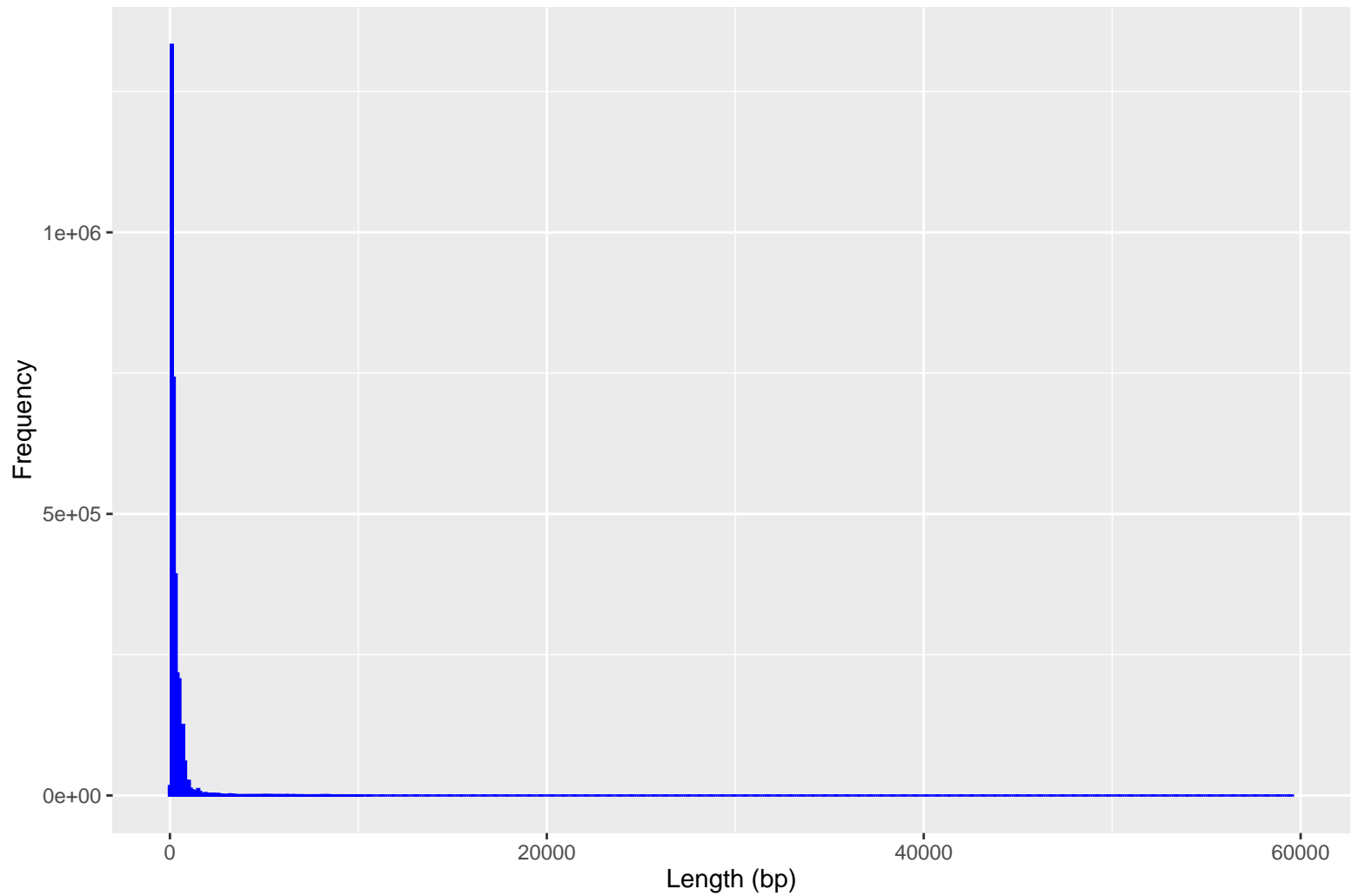

Supplement: giad090_Supplemental_Files [file giad090_supplemental_files.zip › Supplementary Material_Fig. S5.pdf]
