# Supplementary material for: The chromosome-level genome of Chinese praying mantis Tenodera sinensis (Mantodea: Mantidae) reveals its biology as a predator
Source: Gigascience. 2023 Oct 26;12:giad090. doi: 10.1093/gigascience/giad090 (PMC10600911; doi:10.1093/gigascience/giad090)
Supplement: giad090_Response_to_Reviewer_Comments_Original_Submission [file giad090_response_to_reviewer_comments_original_submission.pdf]

Author's Response To Reviewer Comments

Reviewer #1: In this manuscript, Yuan and co-workers report the complete genome assembly of the Chinese praying mantis, Tenodera sinensis. The team used extensive genomic data to reconstruct the genome at the chromosome level, plus they perform a series of exploratory analyses to investigate genomic adaptation, such as gene expansions/contractions of the species.
How the new genomes were sequenced and annotated is sound and according to current standards in the field, making this work a useful resource for community. All the analyses were performed correctly, using correct methodologies. My only concern is related to the phylogenetic analyses, which is performed using a number of distant related species and only ~200 single-copy genes. It's a bit unfortunate because the approach is correct but the number of genes, used to build such a relatively deep phylogeny, is somehow small. I don't think that the small number of genes used may represent a problem for the topology, rather for the estimate of branch lengths and rates for the downstream analyses such as the dating. I'm personally not very surprised that orthofinder was not able to detect enough single-copy genes, but a better approach would be using BUSCO genes. In fact, the mantis genome has a high proportion of single-copy complete genes and the authors could leverage of the them to obtain a more robust set of data. I'm well aware that this would mean rerun some of the downstream analyses, therefore I leave of the authors/editor the decision on re-run the phylogeny.

Response: Due to the limited availability of genomic data for Mantodea and the scarcity of high-quality genomes in polyneoptera, our study focuses on reconstructing phylogenetic relationships on a broader scale. As a result, we obtained a relatively small number of single-copy genes from multiple insect genomes with distant relatives. Our phylogenetic tree constructed using the appropriate methodology aligns with the findings of certain previous studies (e.g., Misof et al., 2014). So we believe this topology can be utilized for further analysis, and therefore, we did not re-run the analysis at present.

Further comments:
Line 21: I'd remove "beneficial insect", not sure there's the need of anthropocentrism for the description of a new insect genome. I think it's valuable enough for the community on its own.

Response: Done. We have deleted the word “beneficial”.

Line 29-30: "We reconfirmed […] within Insecta." The authors should delete the sentence or rephrase is with something like "The reconstructed phylogenetic analysis show the expected topology placing …".

Response: Done. We have revised it.

Line 46: "A fascinating order of insects". I'm not aware of any non-fascinating order of insects.

Response: Done. We have deleted the word “fascinating”.

Line 69-70: "and genetic differences caused by …", I didn't quite understand what the authors mean with that.

Response: Done. What we mean is that there may be genetic diversity in the genomes of different geographic species

Line 86: de novo should be written in italics.

Response: Done.

Line 138: "Using orthofinder, we obtained a total of 216,767 genes".

Response: Done. We have revised it.

Line 141-143: The whole sentence is not clear, what are these 574 unique gene families? Maybe that there are 2436 genes with no orthology relationship clustered in 574 unique OGs?

Response: Done. We have revised it.

Line 153: I'd refer to orthologous groups rather than gene families. I think it's more appropriate.

Response: Done. We have revised it.

Line 188-190: Dating the estimation of effective population size has generally a lot of uncertainty on its own, I personally wouldn't be sure that the correlation to some human activity is also causal effect. I'd be very careful to jump to that conclusions. I'd remove the sentence.

Response: Done. We have deleted it.

Line 194: I'd remove "obviously".

Response: Done. We have deleted it.

Line 340-348: It's a bit confusing. Not sure what "transcriptome sequence was aligned" means in this context. Maybe the authors mean the reads? Also, there's a redundancy in the description of the de novo transcriptome assembly in the previous section (Line 340-342), repeated in the lines 347-348.

Response: Done. We have revised "transcriptome sequence" to "reads", and deleted the redundancy description.

Line 386: I didn't understand the use of Diamond here. Can the authors clarify on that?

Response: DIAMOND is a sequence aligner for protein and translated DNA searches, designed for high performance analysis of big sequence data. Here, we used OrthoFinder to set the parameter "-S diamond" to execute the program.

Line 411-413: I'm not sure that the p value that CAFE returns is per se a sign of fast/rapid evolving OG, rather it should only be associated to the significance of that expansion/contraction statistical confidence.

Response: CAFÉ uses a statistical model to estimate the number of expansion and contraction events of a gene family at that node. The statistical significance of these changes was then assessed by comparing the observed changes in the number of families with the data from the random simulation and calculating a P-value. If the P-value of the change in the number of families at that node is less than a pre-set significance level (P-value < 0.05 were defined as rapidly evolving families in this study), then these gene families are considered to have evolved rapidly at that node.

Reviewer #2: Overall this is a well-presented genome note with another excellent addition to the growing genomic resource, this time for the praying mantis Tenodera sinensis. The underlying data and genome assembly/annotation methods presented are well explained and appear to be sound. The manuscript is generally well-written, but I found some of the conclusions made in the paper to be not particularly well explained in terms of how their data support the various hypotheses being presented. In addition, the Introduction uses quite a lot of "exceptional" language that should be moderated. Some care is needed to either (a) tone down the language or (b) present convincing evidence that support their ideas, or at least explain better how the present data do that. Nonetheless I am happy that the underlying data and genome assembly itself is of high quality and will no doubt be a useful genomic resource going forward.

Response: Many thanks for your positive comments. We have toned down the language in the text as suggested.

Specific comments:

Line 34: why these gene families specifically relating to "predatory digestive demands"

Response: Done. These gene families play a crucial role in the digestive process. Considering that the praying mantis primarily feeds on specific food sources, these gene families contribute significantly to the digestion of the prey within their digestive tracts. But we have removed the word "predatory" because of its inaccuracy and ambiguity.

Line 56: "predation character"—predatory nature?

Response: Done. We have revised it.

Throughout: use of exceptional terminology: "fascinating", "incredible", "unique" etc. E.g. line 48 "each boasting their own unique adaptations and behaviours"—surely this is true for any species?

Response: Done. We have thoroughly revised them in the text.

Line 62:

Response: Done.

Line 64: "fragmented studies"—do you mean studies using fragmented genome assemblies, or just that there have been only a few studies?

Response: Done. We mean the latter and have revised it.

Line 74: "valuable genomic resources for researchers studying predators"—this sounds too broad a claim, I'm not sure the mantis genome would tell us much about mammalian predators, for example… Do you perhaps mean "insect predators" specifically?

Response: Done. We have revised it.

Line 81: why would a "low level" of heterozygosity make it difficult to assemble? I would have thought it would be the other way around, unless you are referring to a fully resolved (diploid) assembly.

Response: Done. We have revised it. Given the large size of the genome, assembling a chromosome-level genome may prove to be challenging.

Line 85: what is meant by "clean data"?

Response: Done. It means the data after quality control.

Line 97: the genome size quoted here (2.48 Gb) is different to that presented in Table 1 (2.54 Gb)?

Response: Done. We have revised it to the correct 2.54 Gb.

Line 99: please provide the total number of genes in the insecta and arthropoda BUSCO gene sets here or in Table 1.

Response: Done. We have provided it in Table 1.

Table 2: typo "Swiss-port" Swiss-prot

Response: Done.

Table 2: it is unclear what the numbers refer to in the "Functional annotation" column—are these numbers

of genes with hits to the various databases? Please explain in the legend.

Response: Done. We have explained it in the legend.

Line 142: what is special about the 2436 genes here? Are these singleton genes unique to T. sinensis, whereas the 574 mentioned previously clustered into a gene family that was unique to T. sinensis?

Response: Done. It means there are 2436 genes with no orthologous relationship clustered in 574 unique OGs, and we have revised it.

Line 165: I'm a bit unconvinced by this. Of course these gene families may play a role in the mantis lifestyle, but is there any direct evidence for adaptive evolution here? It may just be a coincidence (i.e. drift), or driven by some other function unrelated to predation. I suggest toning down the strength of your remarks a little.

Response: Done. We have revised it.

Line 171: for the comparison of genome size, it would be useful to show the genome sizes of a few other species for comparison. ~2.5Gb seems very large for an insect, so it would be nice to see where this was on the distribution of genome sizes for insects/arthropods.

Response: We have considered this possibility and conducted relevant analyses. However, the presence of large genomes in primitive taxa, including Dictyoptera, poses challenges due to their low quality (evidenced by low BUSCO scores, low scaffold N50, and non-chromosome level genomes). The high computational requirements for analyzing these large genomes have also caused difficulties in our analysis. We hope that future research by other scientists can provide a more comprehensive explanation for the occurrence of large genomes in a broader range of insects/arthropods. We remain hopeful that our data can be valuable in the future when such explanations become available.

Line 180: genome duplication? Is the genome not diploid? Do you mean enlargement?

Response: Done. We have revised it.

Line 182: "significantly higher"—is there a statistical test to support this statement?

Response: Done. In the sentence, “other insects” presents other three insects in Dictyoptera that we had analyzed in this part. The mantis showed 18.47% of LTR retrotransposons in the proportion, which is higher than other three insects in Dictyoptera (Figure 4. B). The expression used in the sentence was not precise, and we have made revision accordingly.

Line 194: delete "obviously", I'm not sure this is the correct meaning here.

Response: Done. We have deleted it.

Line 206: typo, delete "great"

Response: Done. We have deleted it.

Line 240: it's not clear how the location of these genes in the genome indicates their role in "intricate diet mechanisms" in this insect? Perhaps a bit more explanation needed.

Response: Done. What we are trying to explain here is that their role in the diet mechanism is represented by "multiple copies", not their location. Due to the average of our writing, which led to the ambiguity, we have revised it.

Line 273: more explanation is needed to explain how a lower rate of Ka/Ks would explain the "peak sensitivity in the 'green' region without other peaks detected in the spectrum".

Response: Done. This sentence is based on the conclusion of "the more LWS opsins were identified" in the previous paragraph, and has nothing to do with “the Ka/Ks of UV-sensitive opsin" in this paragraph, which is a mistake in our writing and has been corrected.

Line 278: was the sequenced individual a male or a female?

Response: Done. We have added the gender description in “Sampling”.

Line 305: what steps were taken to ensure the final assembly did not contain any sequences from non-target organisms deriving from contaminants/co-symbionts in the DNA libraries? I'm sure the data are clean but it would be good to indicate that this check has been done (e.g., BlobTools).

Response: Done. We have aligned the final assembly to the NT library and counted the result to ensure that the final assembly does not contain any sequences of non-target organisms from contaminants/symbionts in the DNA library.

Line 387: only 221 single-copy genes? this seems very small, any ideas why?

Response: Due to the limited availability of genomic data for Mantodea and the scarcity of high-quality genomes in polyneoptera, our study focuses on reconstructing phylogenetic relationships on a broader scale, encompassing Insecta as a whole. As a result, we have obtained a relatively small number of single-copy genes from multiple insect genomes with distant relatives. However, despite this limitation, our phylogenetic tree constructed using the appropriate methodology aligns with the findings of certain previous studies (e.g., Misof et al., 2014). We believe that this topology can be utilized for further analysis.

Line 391: typo "proteins"

Response: Done.

--
