# Supplementary material for: The chromosome-level genome of Chinese praying mantis Tenodera sinensis (Mantodea: Mantidae) reveals its biology as a predator
Source: Gigascience. 2023 Oct 26;12:giad090. doi: 10.1093/gigascience/giad090 (PMC10600911; doi:10.1093/gigascience/giad090)
Supplement: giad090_Reviewer_1_Report_Original_Submission — Francesco Cicconardi -- 7/22/2023 Reviewed [file giad090_reviewer_1_report_original_submission.pdf]

Reviewer Report

Title: The chromosome-level genome of Chinese praying mantis Tenodera sinensis (Mantodea: Mantidae) reveals its biology as a predator

Version: Original Submission **Date:** 7/22/2023

Reviewer name: Francesco Cicconardi

Reviewer Comments to Author:

In this manuscript, Yuan and co-workers report the complete genome assembly of the Chinese praying mantis, Tenodera sinensis. The team used extensive genomic data to reconstruct the genome at the chromosome level, plus they perform a series of exploratory analyses to investigate genomic adaptation, such as gene expansions/contractions of the species.How the new genomes were sequenced and annotated is sound and according to current standards in the field, making this work a useful resource for community. All the analyses were performed correctly, using correct methodologies. My only concern is related to the phylogenetic analyses, which is performed using a number of distant related species and only ~200 single-copy genes. It's a bit unfortunate because the approach is correct but the number of genes, used to build such a relatively deep phylogeny, is somehow small. I don't think that the small number of genes used may represent a problem for the topology, rather for the estimate of branch lengths and rates for the downstream analyses such as the dating. I'm personally not very surprised that orthofinder was not able to detect enough single-copy genes, but a better approach would be using BUSCO genes. In fact, the mantis genome has a high proportion of single-copy complete genes and the authors could leverage of the them to obtain a more robust set of data. I'm well aware that this would mean rerun some of the downstream analyses, therefore I leave of the authors/editor the decision on re-run the phylogeny.Further comments:Line 21: I'd remove "beneficial insect", not sure there's the need of anthropocentrism for the description of a new insect genome. I think it's valuable enough for the community on its own.Line 29-30: "We reconfirmed […] within Insecta." The authors should delete the sentence or rephrase is with something like "The reconstructed phylogenetic analysis show the expected topology placing …".Line 46: "A fascinating order of insects". I'm not aware of any non-fascinating order of insects.Line 69-70: "and genetic differences caused by …", I didn't quite understand what the authors mean with that.Line 86: de novo should be written in italics.Line 138: "Using orthofinder, we obtained a total of 216,767 genes".Line 141-143: The whole sentence is not clear, what are these 574 unique gene families? Maybe that there are 2436 genes with no orthology relationship clustered in 574 unique OGs?Line 153: I'd refer to orthologous groups rather than gene families. I think it's more appropriate.Line 188-190: Dating the estimation of effective population size has generally a lot of uncertainty on its own, I personally wouldn't be sure that the correlation to some human activity is also causal effect. I'd be very careful to jump to that conclusions. I'd remove the sentence.Line 194: I'd remove "obviously".Line 340-348: It's a bit confusing. Not sure what "transcriptome sequence was aligned" means in this context. Maybe the authors mean the reads? Also, there's a redundancy in the description of the de novo transcriptome assembly in the previous section (Line 340-342), repeated in the lines 347-348.Line 386: I didn't understand the use of Diamond here. Can

the authors clarify on that?Line 411-413: I'm not sure that the p value that CAFE returns is per se a sign of fast/rapid evolving OG, rather it should only be associated to the significance of that expansion/contraction statistical confidence.

Level of Interest

Please indicate how interesting you found the manuscript: Choose an item.

Quality of Written English

Please indicate the quality of language in the manuscript: Choose an item.

Declaration of Competing Interests

Please complete a declaration of competing interests, considering the following questions:

- Have you in the past five years received reimbursements, fees, funding, or salary from an organisation that may in any way gain or lose financially from the publication of this manuscript, either now or in the future?
- Do you hold any stocks or shares in an organisation that may in any way gain or lose financially from the publication of this manuscript, either now or in the future?
- Do you hold or are you currently applying for any patents relating to the content of the manuscript?
- Have you received reimbursements, fees, funding, or salary from an organization that holds or has applied for patents relating to the content of the manuscript?
- Do you have any other financial competing interests?
- Do you have any non-financial competing interests in relation to this paper?

If you can answer no to all of the above, write 'I declare that I have no competing interests' below. If your reply is yes to any, please give details below.

I declare no competing interests for all the above

I agree to the open peer review policy of the journal. I understand that my name will be included on my report to the authors and, if the manuscript is accepted for publication, my named report including any attachments I upload will be posted on the website along with the authors' responses. I agree for my report to be made available under an Open Access Creative Commons CC-BY license (http://creativecommons.org/licenses/by/4.0/). I understand that any comments which I do not wish to be included in my named report can be included as confidential comments to the editors, which will not be published.

Choose an item.

To further support our reviewers, we have joined with Publons, where you can gain additional credit to further highlight your hard work (see: https://publons.com/journal/530/gigascience). On publication of this paper, your review will be automatically added to Publons, you can then choose whether or not to claim your Publons credit. I understand this statement.

Yes Choose an item.
