# Supplementary material for: The chromosome-level genome of Chinese praying mantis Tenodera sinensis (Mantodea: Mantidae) reveals its biology as a predator
Source: Gigascience. 2023 Oct 26;12:giad090. doi: 10.1093/gigascience/giad090 (PMC10600911; doi:10.1093/gigascience/giad090)
Supplement: giad090_Reviewer_2_Report_Original_Submission — Reuben Nowell -- 7/26/2023 Reviewed [file giad090_reviewer_2_report_original_submission.pdf]

Reviewer Report

Title: The chromosome-level genome of Chinese praying mantis Tenodera sinensis (Mantodea: Mantidae) reveals its biology as a predator

Version: Original Submission **Date:** 7/26/2023

Reviewer name: Reuben Nowell

Reviewer Comments to Author:

Overall this is a well-presented genome note with another excellent addition to the growing genomic resource, this time for the praying mantis Tenodera sinensis. The underlying data and genome assembly/annotation methods presented are well explained and appear to be sound. The manuscript is generally well-written, but I found some of the conclusions made in the paper to be not particularly well explained in terms of how their data support the various hypotheses being presented. In addition, the Introduction uses quite a lot of "exceptional" language that should be moderated. Some care is needed to either (a) tone down the language or (b) present convincing evidence that support their ideas, or at least explain better how the present data do that. Nonetheless I am happy that the underlying data and genome assembly itself is of high quality and will no doubt be a useful genomic resource going forward.Specific comments:Line 34: why these gene families specifically relating to "predatory digestive demands"Line 56: "predation character"â€"predatory nature?Throughout: use of exceptional terminology: "fascinating", "incredible", "unique" etc. E.g. line 48 "each boasting their own unique adaptations and behaviours"â€"surely this is true for any species?Line 62:Line 64: "fragmented studies"â€"do you mean studies using fragmented genome assemblies, or just that there have been only a few studies?Line 74: "valuable genomic resources for researchers studying predators"â€"this sounds too broad a claim, I'm not sure the mantis genome would tell us much about mammalian predators, for example… Do you perhaps mean "insect predators" specifically?Line 81: why would a "low level" of heterozygosity make it difficult to assemble? I would have thought it would be the other way around, unless you are referring to a fully resolved (diploid) assembly.Line 85: what is meant by "clean data"?Line 97: the genome size quoted here (2.48 Gb) is different to that presented in Table 1 (2.54 Gb)?Line 99: please provide the total number of genes in the insecta and arthropoda BUSCO gene sets here or in Table 1.Table 2: typo "Swiss-port" > Swiss-protTable 2: it is unclear what the numbers refer to in the "Functional annotation" columnâ€"are these numbers of genes with hits to the various databases? Please explain in the legend.Line 142: what is special about the 2436 genes here? Are these singleton genes unique to T. sinensis, whereas the 574 mentioned previously clustered into a gene family that was unique to T. sinensis?Line 165: I'm a bit unconvinced by this. Of course these gene families may play a role in the mantis lifestyle, but is there any direct evidence for adaptive evolution here? It may just be a coincidence (i.e. drift), or driven by some other function unrelated to predation. I suggest toning down the strength of your remarks a little.Line 171: for the comparison of genome size, it would be useful to show the genome sizes of a few other species for comparison. ~2.5Gb seems very large for an insect, so it would be nice to see where this was on the distribution of genome sizes for insects/arthropods.Line 180: genome duplication? Is the genome not diploid? Do you mean

enlargement?Line 182: "significantly higher"â€”is there a statistical test to support this statement?Line 194: delete "obviously", I'm not sure this is the correct meaning here.Line 206: typo, delete "great"Line 240: it's not clear how the location of these genes in the genome indicates their role in "intricate diet mechanisms" in this insect? Perhaps a bit more explanation needed.Line 273: more explanation is needed to explain how a lower rate of Ka/Ks would explain the "peak sensitivity in the 'green' region without other peaks detected in the spectrum".Line 278: was the sequenced individual a male or a female?Line 305: what steps were taken to ensure the final assembly did not contain any sequences from non-target organisms deriving from contaminants/co-symbionts in the DNA libraries? I'm sure the data are clean but it would be good to indicate that this check has been done (e.g., BlobTools).Line 387: only 221 single-copy genes? this seems very small, any ideas why?Line 391: typo "proteins"

Level of Interest

Please indicate how interesting you found the manuscript: Choose an item.

Quality of Written English

Please indicate the quality of language in the manuscript: Choose an item.

Declaration of Competing Interests

Please complete a declaration of competing interests, considering the following questions:

- Have you in the past five years received reimbursements, fees, funding, or salary from an organisation that may in any way gain or lose financially from the publication of this manuscript, either now or in the future?
- Do you hold any stocks or shares in an organisation that may in any way gain or lose financially from the publication of this manuscript, either now or in the future?
- Do you hold or are you currently applying for any patents relating to the content of the manuscript?
- Have you received reimbursements, fees, funding, or salary from an organization that holds or has applied for patents relating to the content of the manuscript?
- Do you have any other financial competing interests?
- Do you have any non-financial competing interests in relation to this paper?

If you can answer no to all of the above, write 'I declare that I have no competing interests' below. If your reply is yes to any, please give details below.

I declare that I have no competing interests

I agree to the open peer review policy of the journal. I understand that my name will be included on my report to the authors and, if the manuscript is accepted for publication, my named report including any

attachments I upload will be posted on the website along with the authors' responses. I agree for my report to be made available under an Open Access Creative Commons CC-BY license (http://creativecommons.org/licenses/by/4.0/). I understand that any comments which I do not wish to be included in my named report can be included as confidential comments to the editors, which will not be published.

Choose an item.

To further support our reviewers, we have joined with Publons, where you can gain additional credit to further highlight your hard work (see: https://publons.com/journal/530/gigascience). On publication of this paper, your review will be automatically added to Publons, you can then choose whether or not to claim your Publons credit. I understand this statement.

Yes Choose an item.
